# Vaccination for the Prevention of Neonatal Calf Diarrhea in Cow-Calf Operations: A Scoping Review

**DOI:** 10.1016/j.vas.2022.100238

**Published:** 2022-02-19

**Authors:** Gabriele Ute Maier, Jefferson Breitenbuecher, Jose Pablo Gomez, Festus Samah, Erik Fausak, Megan Van Noord

**Affiliations:** a Department of Population Health and Reproduction, School of Veterinary Medicine, University of California Davis, 1 Shields Ave, Davis, CA, 95616, USA; b University Library, University of California, Davis, 1 Shields Ave, Davis, CA, 95616, USA

**Keywords:** Calf scours, diarrhea, enteritis, vaccine, Abbreviations, CDFA-AUS California Department of Food and Agriculture–Antimicrobial Use and Stewardship, SYREAF Systematic Reviews for Animals and Food, PRISMA preferred reporting items for systematic review and meta-analysis protocols

## Abstract

Neonatal calf diarrhea (NCD), also known as scours, is an important disease of preweaned calves that affects the production and welfare of beef herds. While hygiene and nutrition are important in reducing the incidence of NCD, vaccination of dams or calves is often employed for the prevention of NCD. The present scoping review summarizes the available peer-reviewed scientific English literature on vaccination of dams or calves for the prevention of NCD over the past decades. The online databases Medline, CAB Abstracts, and Biosis were searched for articles on the topic published between 1950 and 2020. Online software was used to systematically evaluate 2807 citations for inclusion through pre-determined criteria in a 2-step process. In the 113 articles included in the review, vaccines tested targeted the pathogens *E. coli* (n = 43), bovine rotavirus (BRV, n = 38), *Salmonella* (n = 29), bovine coronavirus (BCV, n = 14), bovine viral diarrhea virus (n= 7), and other pathogens (n = 8). Field trials for commercial vaccines have been published for the most important pathogens, and results on efficacy are variable for such vaccines targeting BRV, BCV, and *E. coli.* Meta-analyses exploring efficacy of these vaccines would be helpful to practitioners and producers. No field studies on commercial products have shown efficacy for *Salmonella* vaccines so that a meta-analysis would unlikely come to a different conclusion. Further research is needed on vaccines for protozoal pathogens like *Cryptosporidium parvum* as well as on the importance of several emerging enteric viruses in calves.

## Highlights

• Since 1950, 113 articles on vaccines for the prevention of neonatal calf diarrhea have been published in the English literature
• Results for field trials using commercial vaccines for *E. coli*, bovine rotavirus, and bovine coronavirus infections are variable
• No field trials for commercial *Salmonella* vaccines have shown efficacy
• Vaccines for protozoal pathogens causing calf scours as well as the importance of several emerging enteric viruses of cattle need further research

## 1 Introduction

Neonatal calf diarrhea (NCD), also known as calf scours or enteritis, is a gastrointestinal disease affecting pre-weaned calves. The disease can be fatal to young calves as a consequence of hypovolemia and acidosis that may also result in anorexia and ataxia.

Neonatal calf diarrhea can be economically devastating to producers due to high mortality and impaired growth of affected animals (de Graaf et al., 1999; Hasler et al., 2012). In cow-calf operations in the U.S. in 2007, losses from digestive problems made up 14% of deaths in calves less than three weeks old and 23% of deaths in calves three weeks of age and older (USDA, 2010).

The disease can be attributed to both infectious and non-infectious causes. Non-infectious causes of NCD include vitamin deficiency or abrupt changes in diet, or errors in milk replacer feeding, while infectious causes involve a variety of pathogens including viral, protozoal, and bacterial pathogens, where one or more can be involved in the disease process (Cho et al., 2013). Diagnosis of the disease-causing pathogen requires laboratory examination of samples from diseased animals (Cho & Yoon, 2014).

The standard treatment for scouring calves includes oral or intravenous fluid therapy, isolation of infected animals, supportive care including anti-inflammatories and may include the use of antimicrobials (Constable, 2009; Smith & Berchtold, 2014). Antimicrobial treatment of calf diarrhea is, however, controversial. While opponents of antimicrobial use in the treatment of NCD state the potential for the emergence of antimicrobial resistant bacteria, proponents argue that a proportion of calves with diarrhea are bacteremic and are at risk of developing septicemia, and could therefore benefit from antimicrobial treatment (Smith, 2015).

Methods recommended for the prevention of NCD include proper cow nutrition during pregnancy, dystocia management, reduction of environmental stress and contamination, and ensuring transfer of passive immunity to the calf via colostrum, enhancing the presence of specific antibodies in the gut lumen during the calf's most vulnerable age for NCD (Snodgrass, 1986), and bolstering the calf's immunity via systemic uptake of antibodies (Al-Alo et al., 2018; Cho & Yoon, 2014). Colostrum quality depends partly on vaccination of the cow during pregnancy (Geletu et al. 2021, Meganck et al., 2014, Menichetti et al. 2021). Currently, commercial vaccines for the most important pathogens that cause calf diarrhea are available and are intended for vaccination of either the dam or the calf.

The California Department of Food and Agriculture's Antimicrobial Use and Stewardship group (CDFA-AUS) conducts a statewide program to promote the judicious use of antimicrobials in livestock species and poultry with the goal of preserving efficacy of these essential drugs as well as protecting public health through measures that minimize the emergence of antimicrobial resistant bacteria. A 2016 survey of cow-calf producers in California showed that NCD is one of three diseases that was most frequently treated with antimicrobials by respondents (CDFA-AUS, 2020). Although recommendations for prevention methods such as vaccination are available in the form of review papers on the topic, we are not aware of any scoping reviews or meta-analyses on the efficacy of vaccines for NCD.

The objective of this study was to provide a comprehensive overview of the literature on studies of vaccines to prevent NCD relevant to California cow-calf operations. We aimed to identify the existing literature and describe vaccines used and outcomes reported. Further objectives were to identify the need for meta-analyses in specific areas with abundant information or further research in areas with a lack of information.

The resulting scoping review is part of the effort by CDFA-AUS to develop best practices for California cow-calf operations. It is expected that the usefulness of the resulting tools and documents will expand beyond the state boundaries. Although the scoping review was conducted with practices and conditions in California in mind, the information gained may be applicable to a much broader audience.

## 2 Materials and Methods

### 2.1 Protocol and Registration

The preferred reporting items for systematic review and meta-analysis protocols (PRISMA-P) (Liberati et al., 2009) as well as the PRISMA Extension for Scoping Review guidelines (Tricco et al., 2018) were used as references to conduct the review. An a priori review protocol was developed and archived in eScholarship, the online repository of the University of California (Maier, 2020) and was registered with Systematic Reviews for Animals and Food (SYREAF) (http://www.syreaf.org).

### 2.2 Focus Group Engagement

Before study start, in May 2019, a group of stakeholders consisting of bovine practitioners in private practice, academia, and from the state animal health regulatory body, a veterinary laboratory diagnostician, and an animal science faculty member were invited to an in-person meeting to discuss the objectives and scope of the review. Topics discussed during the focus group meeting were inclusion criteria, such as timeframe, geographic limitations, allowable study designs, relevant study populations, outcomes and interventions, and which pathogens to consider to best meet the objective of creating science-based guidelines applicable to cow-calf producers in helping them prevent NCD.

### 2.3 Eligibility Criteria

Based on the focus group discussion, original scientific reports in the form of peer reviewed research studies or conference proceeding of ≥ 500 words published in English language in or after 1950 involving outcomes in calves under 6 months of age belonging to the genus *Bos* at the individual or herd level were eligible for inclusion in the review. All observational or experimental study designs were included except case studies or case series. No studies were excluded based on geographical study location.

Studies had to compare a vaccination regime for the prevention of NCD to either placebo or another intervention and have a clinical quantifiable outcome beyond immune responses including but not limited to disease incidence, disease-specific mortality, duration of disease, or weight gain. Studies had to include a diagnosis of any of the causative agents bovine rotavirus (BRV), bovine coronavirus (BCV), bovine viral diarrhea virus (BVDV), torovirus, norovirus, nebovirus, *Salmonella, E. coli, Clostridium perfringens, Shigella, Yersinia, Cryptosporidium*, or *Giardia*, or a clinical diagnosis of diarrhea. Studies had to be applicable to cow-calf operations, however, this did not necessarily preclude studies conducted on dairies or in dairy breeds, as the principles of immunology apply to both production settings. In the broadest terms, the question was whether the intervention could be implemented as described or in a modified way by producers in the daily operation of a cow-calf herd in terms of invasiveness of procedures or applicability to beef production, as opposed to dairy or calf ranch environments.

### 2.4 Sources of Information

The database search was designed and conducted through the Carlson Health Library at the University of California, Davis. The databases Medline (Pubmed interface, 1966 to 2020), CAB Abstracts (CAB Direct interface, 1972 to 2020) and Biosis (Web of Science interface, 1926 to 2020) were searched on November 22, 2019, and results combined in the reference manager software Endnote (Endnote, Clarivate Analytics, Philadelphia, Pennsylvania) where duplicates were deleted. The search strategy employed terms describing the population, disease, pathogens, and intervention with restrictions on language and publication date. Keywords from key references were collected and compared with the keywords utilized previously. Yale MeSH Analyzer (http://mesh.med.yale.edu/) was also utilized to compare common Medical Subject Headings across articles. The full electronic search strategy for Medline, CAB Abstracts, and Biosis including limits used, is available in Appendix A as well as in the study protocol (Maier, 2020).

Conference proceedings and vaccine manufacturers’ unpublished studies were searched and evaluated as part of the grey literature. Conference proceedings for the American Association of Bovine Practitioners, the World Buiatrics Association, American College of Veterinary Internal Medicine, and the Conference of Research Workers in Animal Diseases, were extracted from S-Pac (Searchable Proceedings of Animal Conferences) and citations hand searched for suitable inclusions into the review. The webpages of NCD vaccine manufacturers were searched for studies that were not included in the above search and their technical services contacted where possible to inquire about any additional studies.

Finally, as an additional quality control step, articles included in the data extraction level of the review underwent a search through the Scopus database (www.Scopus.com) for their citations and those that cited these articles. Results of the Scopus search were compared to articles already identified in the previous database searches and any new articles hand selected for inclusion into the review.

### 2.5 Study selection and data extraction

After importation of study references into the systematic review online software platform DistillerSR (Evidence Partners, Ottawa, Canada) and an additional de-duplication step, the remaining studies underwent a two-level screening by two reviewers who independently assessed the relevance of studies by title and abstract and then by full text using predefined eligibility questions based on PICOS (Population - Intervention - Comparison – Outcome – Study type) elements of eligibility criteria (Schardt et al., 2007).

The questions to pass two levels of screening were as follows: Is the full text available in English? Is the study a case report or case series? Does the study compare vaccination regimes for the prevention of scouring, diarrhea, or enteritis in pre-weaned calves or calves 6 months old or younger? Is there a concurrent comparison group? Does the study make a diagnosis of scours/diarrhea/enteritis based on the pathogen (viral, protozoal, or bacterial) or a clinical diagnosis? Can the intervention be generalized to cow-calf operations in California (not only specific for dairies or calf ranches)? Does the study report a quantifiable outcome to evaluate the efficacy (e.g. incidence of diarrheic calves, cause specific mortality due to gastrointestinal disease)? Is the study published in a peer-reviewed journal or conference proceedings ≥ 500 words?

Studies were excluded if both reviewers responded “no” to any of the questions, except to the question “Is this study a case report or case series”, to which reviewers had to answer “yes” to exclude the study. Disagreements were resolved by a third reviewer. Screening questions were pre-tested on a random selection of twenty studies included in the first level and ten studies included in the second level by reviewers to validate screening questions and reach consensus on wording and interpretation of criteria. Data extraction from each study that passed the screening steps was performed by one reviewer in DistillerSR. The type of data extracted from each study is described in Table 1. Data was exported to Microsoft Excel and analyzed in R studio (version 1.2.5019).

**Table 1:** Description of data charting items for relevant journal articles or proceedings for a scoping review on vaccination for the prevention of neonatal calf diarrhea in cow-calf operations

Variable	Description of information extracted
Study characteristics	Year of publication, region and country where study was performed (North America, Central America, South America, Europe, Asia, Africa, Australia/New Zealand), publication type (Peer reviewed journal, conference abstract), study population: production system (beef, dairy, not stated), calf age group (preweaned, weaned), breed (Angus, Hereford, Holstein, etc.), sex (female, steers, bulls, not stated), herd type (commercial, research, not stated), housing type (pasture, barn, laboratory, etc.), study type (experimental, observational), vaccine recipient (dam, calf), when was vaccine given, route of administration (intramuscular, subcutaneous, oral, etc.), for experimental studies: randomized group allocation, control group type (placebo, no intervention, different intervention), researchers blinded to group allocation, how was diagnosis established (culture, clinical diagnosis)
Study outcomes	Disease incidence, disease severity (severity score, duration of illness), weight gain, disease specific mortality, risk ratio
Study groups	Verbal description of group (vaccinated, control, etc.), how many animals in the group
Intervention per group	Type of vaccine studied (none (for controls), modified live, killed, toxoid, subunit/conjugate, recombinant, etc)
Results per study group	Intervention statistically improved outcome between study groups, statistically worsened outcome between study groups, statistically did not change outcome between groups, or statistical results not provided

## 3 Results

### 3.1 Sources of studies

**Fig. 1:**
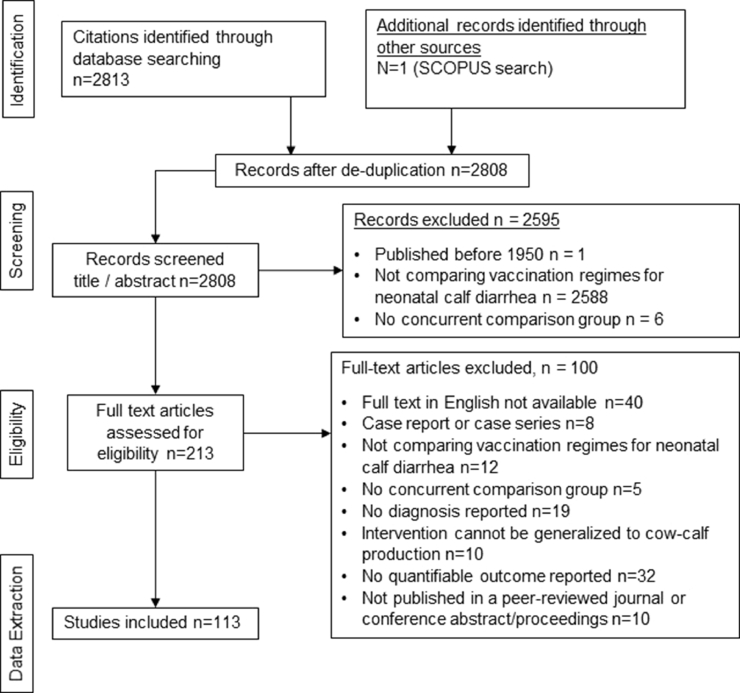
Scoping review on vaccination for the prevention of neonatal calf diarrhea: PRISMA flow diagram of citations form the literature search to relevance screening and data extraction.

There were 2807 unique citations identified through the initial literature searches including grey literature. An additional 1682 unique citations were identified following the SCOPUS search of data-charted articles. After manually scanning through articles identified in the SCOPUS search published during the last 20 years (since the year 2000) one additional reference was added to the review for a total of 2808 references. Vaccine manufacturers did not provide any additional studies. There were 213 (7.6 %) articles that underwent a full text review for eligibility with 113 (4.0 %) undergoing data extraction (Figure 1). Citations for all 113 studies are listed in Appendix B categorized by targeted pathogen.

### 3.2 Study characteristics

**Fig. 2:**
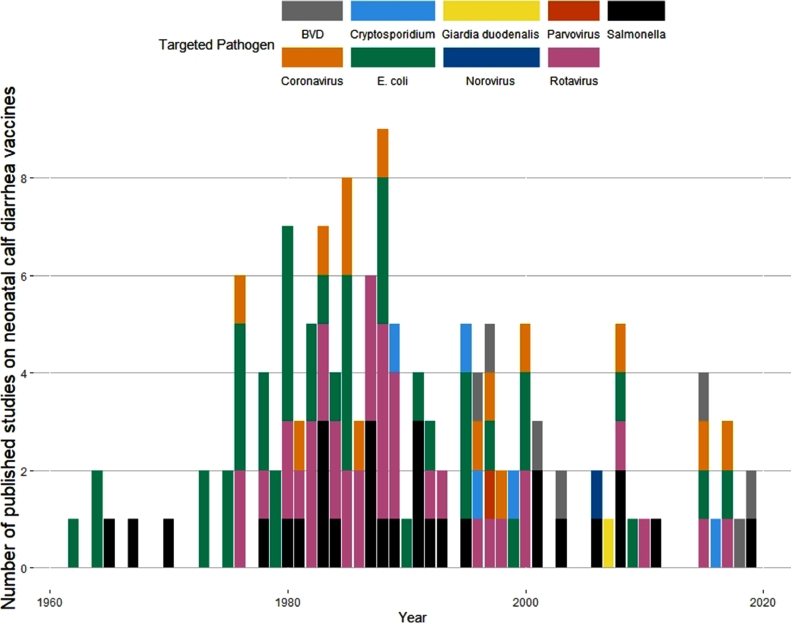
Scoping review on vaccination for the prevention of neonatal calf diarrhea: number of studies published per year between 1960 and 2020 color-coded by target pathogen. Studies with multivalent vaccines are counted multiple times.

Study characteristics including pathogens targeted, vaccine recipient, vaccine type, region where study was performed, study size, breed type, and study design are summarized in Table 2. Study sizes were recorded as number of calves in the study, i.e., when dams were vaccinated, they were not counted. The number of calves in three studies could not be determined, because the study design only described number of herds enrolled, and these studies are not included in the calculation of the total study size parameters in table 2. The number of publications for the different target pathogens per year is depicted in Figure 2. Peer reviewed journal publications made up 109 articles, while three were conference abstracts of 500 words or more and one was a from a chapter of studies published in book format. While the majority of studies were performed in preweaned calves, eight publications mentioned the use of weaned calves only, and two used both weaned and preweaned calves. Predominantly beef breeds were used in 14 studies including the breeds Angus, Hereford, Angus/Charolais mix, Japanese Black, native Korean, Slovak Red, Gascon, Limousin, Aubrac, and Blonde Aquitaine. Predominantly dairy breeds were mentioned in 54 articles, including Holstein, Friesian, Jersey, Brown Swiss, Ayrshire, Guernsey, and various crosses of those breeds. Dairy/beef crosses including Friesian/Hereford crossbred cattle were mentioned in three articles. No specific breed of cattle was mentioned in 42 articles. Sex of study calves was not mentioned in 78 articles, 19 used only male calves, 2 used only female calves, and 14 had both male and female calves in their studies. Commercial herds were used in 46 articles, research herds were used in 47 articles, both research and commercial herds were mentioned in 3 articles, and in 17 studies we could not determine what the herd type was. Barns were used as housing type in 3 studies, 46 studies mentioned individual pens, boxes, isolation facilities with individual pens, or a laboratory or experimental type setting as the housing type during the study period, 13 articles mentioned studies that were carried out on pasture, and 51 did not specifically state what the housing or environment was. Randomization to study groups was mentioned in 28 articles, blinding of researchers to group allocation was described in 10 articles. Combination vaccines covering more than one pathogen were studied in 19 articles.

**Table 2:** Counts of published literature for different study design characteristics, across three time periods, identified in a scoping review on vaccination for the prevention of neonatal calf diarrhea relevant to cow-calf operations.

		Years		
Characteristic	pre 1980	1980-1989	1990-2020	Total
Pathogen targeted				
E. coli	14	15	14	43
Salmonella	4	11	14	29
Bovine rotavirus	3	24	11	38
Bovine coronavirus	1	6	7	14
Bovine viral diarrhea virus	0	0	7	7
Other	0	1	7	8
Vaccine recipient				
Dam	14	34	20	68
Calf	6	14	25	45
Vaccine type				
Commercial	3	15	18	36
Experimental	17	33	27	77
Region				
North America	13	17	20	50
Australia/New Zealand	0	1	4	5
Europe	7	26	8	41
Africa	0	0	2	2
Asia	0	3	6	9
South America	0	1	5	6
Total study size				
Median (IQR)	51 (40, 307)	43 (22, 110)	29 (16, 101)	41 (23, 132)
Mean, (SD)	563 (1149)	497 (1386)	308 (781)	435 (1134)
Range	10 - 4068	8 - 6787	6 - 4053	6 – 6787
Breed Type				
Beef	8	4	6	18
Dairy	0	20	28	48
Beef & Dairy	2	2	0	4
Not Stated	10	21	11	42
Dairy & Not Stated	0	1	0	1
Study Design				
Experimental	20	47	40	107
Observational	0	1	5	6
Challenge Type				
Natural	7	12	17	36
Experimental	13	30	28	71
Both^1^	0	6	0	6

1 Articles in this category contained trials with both natural and experimental challenge.

### 3.3 Individual pathogens

#### 3.3.1 Bovine rotavirus

Bovine rotavirus is in the family of Reoviridae and named Reovirus in earlier studies. We counted studies on reovirus vaccines together with the BRV vaccines. Timing of the first vaccination given to calves ranged between the first 6 hours to the first 3 days of life, with only 5 studies vaccinating calves, while 34 studies relied on vaccination of the dams for calf protection from BRV. Timing of dam vaccination varied widely between studies and consisted in many of the studies of two doses during the last two months of pregnancy. However, in some studies, dam vaccination was performed with wider intervals, e.g. one article mentioned vaccination prior to mating and seven months later approximately two to three months before calving (Snodgrass et al., 1980). Killed vaccines were used in 22 articles, modified live vaccines in 14 articles, one article used a recombinant BRV vaccine, and one used both a modified live and killed vaccine. While 5 of the articles used beef breeds or herds to study BRV vaccines, 3 used beef and dairy cattle, 19 only dairy cattle and 11 did not state the production type. There were 16 studies involving commercial vaccines for BRV, 10 of which studied combination vaccines and 6 that studied vaccines for BRV alone. Results for 11 field trials for commercial BRV vaccines are summarized in Table 3. Of the 11 field trials with commercial BRV vaccines, 3 reported statistically significantly improved outcomes in calves of vaccinated dams, while 3 did not provide statistical results. The remaining studies either saw no differences in outcomes between groups or found undesirable outcomes in groups receiving BRV vaccine with one reporting higher case fatality in calves vaccinated for BRV and dams receiving a placebo compared to those calves receiving a placebo, or where the dam had received an *E. coli* bacterin additionally to calves receiving the BRV vaccine (Acres and Radostits 1976). Another study found a higher number of calves treated for all diseases in vaccinated herds versus unvaccinated herds (Waltner-Toews et al. 1985).

**Table 3:** Scoping review on vaccines for neonatal calf diarrhea: results of field trials for commercial bovine rotavirus and bovine coronavirus vaccines with natural challenge

**Author(s), year**	**Vaccine name, vaccine type, pathogens, route, recipient**	**Study Group name (number in group)**	**Outcome**	**Result**
Acres & Radostits, 1976	Scourvax-Reo, modified live, BRV, oral, calf	1. *E. coli* bacterin to cows, modified live BRV to calves (247)	Incidence of calf diarrhea, diarrhea-specific mortality	No significant differences
		2. *E. coli* bacterin to cows, placebo to calves (268)	Case fatality	Statistically higher in group 3 (placebo to cows, modified live BRV vaccine to calves)
		3. Placebo to cows, modified live BRV vaccine to calves (217)		
		4. Placebo to cows, placebo to calves (264)		
de Leeuw et al., 1980	Scourvax-reo 1, modified live, BRV, BCV, oral, calf	1. Vaccinated (74)	Incidence of BRV-associated diarrhea, Severity score of BRV-associated diarrhea, Incidence of undifferentiated diarrhea, Severity score of undifferentiated diarrhea	No statistics provided; BRV excretion: 20/74 in vaccinated, 19/76 in placebo; severity score of BRV associated diarrhea: 3.0 in vaccinated versus 1.5 in placebo on farm A and 1.0 in vaccinated versus 1.1 in placebo on farm C (higher score = more severe).
		2. Placebo (76)		
Hudson, 1981	Calf Guard, modified live, BRV, BCV, intramuscular, dam	1. Vaccinated (3879)	Incidence of calf diarrhea, case fatality	Statistically lower in vaccinated
		2. Non-vaccinated (1891)	Number of treatments required for diarrhea	No statistics provided: 0.77% (30/3879) of vaccinated requiring two or more treatments versus 3.91% (74/1891) of controls
Bürki et al., 1983	Calf Guard, modified live BRV, BCV oral, calf	1. Vaccinated day 1 (31)	Incidence of diarrhea, severity of diarrhea	No statistics provided
		2. Vaccinated day 3 (24)		
		3. Unvaccinated control (4)		
Waltner-Toews et al., 1985	Scourguard3, modified live BRV, BCV, K99 bacterin, intramuscular, dam	1. Calves of vaccinated cows, trial A (182)	Treatment for diarrhea, mortality, days to first treatment or duration of treatment all diseases, days to first treatment or duration of treatment for diarrhea, estimated weight gain (trial A)	No significant differences
		2. Calves of placebo cows, trial A (95)	Calves treated for all diseases or treated for scours or treated for scours from day 5 – 14, mortality (trial B)	No significant differences
		1. Vaccinated herds, trial B (11 herds, n not stated)	Calves treated for all diseases from day 5 – 14 (trial B)	Statistically higher in vaccinated herds
		2. Non-vaccinated herds, trial B (12 herds, n not stated)		
McNulty & Logan, 1987	Rotavec K99, killed, BRV, *E. coli* K99, intramuscular, dam	1. Vaccinated (19)	Incidence of abnormal feces or diarrhea	No statistics provided; 1/19 in vaccinated versus 9/29 unvaccinated
		2. Unvaccinated (29)		
Kohara et al., 1997	Lactovac, killed, BRV, BCV, Parvovirus, *E. coli* K99 pilus, subcutaneous, dam	1. Vaccinated (48)	Incidence of diarrhea, mean age of onset, duration of diarrhea, mortality	No significant differences
		2. Unvaccianted (48)		
Le Rousic et al., 2000	Lactovac, killed BRV, BCV, *E. coli* K99/F41 antigen, subcutaneous, dam	1. Vaccinated (12)	Severity score of BRV associated diarrhea	Statistically lower in vaccinated
		2. Unvaccinated (12)	Weight gain	No significant difference
Perk et al., 2000	Rotavac, killed, *E. coli K99* and BRV, route not stated, dam	1. Vaccinated with Rotavac, trial 1 (190)	Morbidity, mortality, time to diarrhea, days of diarrhea, severity of diarrhea, number of treatments, trial 1	No statistics provided; mortality in Rotavac group (16/190) versus Nobivac group (18/65). No numbers provided for other outcomes
		2. Vaccinated with Nobivac (*E. coli* K99), trial 1 (65)	Morbidity, mortality, time to diarrhea, days of diarrhea, severity of diarrhea, number of treatments, trial 2	No significant differences between groups
		3. Vaccinated with Rotavac and 13 days of supplementary hyperimmune K99/RV colostrum, trial 2, n not stated		
		4. Vaccinated with Rotavac and 13 days of placebo, trial 2, n not stated		
Meganck et al., 2014	Rotavec-Corona, killed, BRV, BCV, *E. coli,* intramuscular, dam	1. Vaccinated, calves also treated with oral halofuginone (13 herds, n = 296)	Incidence of diarrhea	Statistically lower in treatment herds
		2. Control, no treatments (11 herds, n = 234)	Duration of diarrhea, mortality	No significant difference
Gomes Rocha et al., 2017	Name not stated, killed, BRV, BCV, *E. coli* K99 bacterin, *Clostridium perfringens* toxoid, intramuscular, dam	1. Vaccinated herd (28 calves)	Diarrheic fecal samples positive for BRV	No statistics provided; diarrheic fecal samples collected that are positive for BRV in vaccinated (6/77) versus unvaccinated (3/74).
		2. Non-vaccinated herd (28 calves)		

#### 3.3.2 Salmonella

Dams were vaccinated in only 4 of the 29 articles on *Salmonella* vaccines, while 25 articles studied vaccinated calves. Study size varied between 8 and 1007 animals. While the majority of 17 studies was conducted in dairy cattle, one study was performed with beef cattle, one with both beef and dairy cattle and 10 did not state the production type. In studies that mentioned calf age at vaccination, calves received their first dose between 1 day and 4 weeks of age. Four studies relied on clinical diagnoses only, while all others performed culture or additional diagnostics to establish a diagnosis of salmonellosis. *Salmonella dublin* was the vaccine serovar in 9 articles, *Salmonella typhimurium* in 14. Two articles included vaccines with both *Salmonella dublin* and *Salmonella typhimurium*, one article studied a *Salmonella saint-paul* serovar vaccine, one a *Salmonella newport* serovar vaccine, one article compared a *Salmonella* serotype Cholerasuis vaccine to both a *Salmonella montevideo* serovar vaccine and control, while one article studied dialyzable leukocyte extract as a *Salmonella* vaccine. The same serovar, although often a different strain, was used to challenge vaccinated animals in 14 of the 22 articles that described a challenge, while in 7 articles a different serovar was used and 1 article used both the same as well as a different serovar from the vaccine serovar, although not in the same group of animals.

Results for three field trials for commercial *Salmonella* vaccines are summarized in Table 4. None of the field trials was able to find any statistically significant differences between vaccinated and control calves in the outcomes they evaluated. One of the field trials used an injectable vaccine in extra-label fashion orally in calves.

**Table 4:** Scoping review on vaccines for neonatal calf diarrhea: results of field trials for commercial *Salmonella* vaccines with natural challenge

**Author, year**	**Vaccine name, vaccine type, pathogens, route, recipient**	**Study Group names (number in group)**	**Outcome**	**Result**
Peters et al., 1987	Bovivac Plus, killed, *Salmonella, E. coli, Pasteurella*, subcutaneous, calf	1. Vaccinated (125)	Morbidity or mortality from all causes	No difference
		2. Unvaccinated (33)		
House et al., 2001	SC-54, modified live, *Salmonella* Cholerasuis, intramuscular, dam	1. Vaccinated commercial vaccine (31)	Incidence of *Salmonella* shedding, mortality	No difference
		2. Vaccinated autogenous bacterin (31)	Frequency of shedding	Statistically significantly lower in vaccinated with commercial vaccine versus autogenous bacterin but not versus unvaccinated
		3. Unvaccinated (18)		
Habing et al., 2011	Entervene-D, modified live, *Salmonella* Dublin, oral, calf	1. Vaccinated (140)	Morbidity from all causes, *Salmonella*-specific morbidity, weight gain, mortality	No difference
		2. Placebo (148)		

#### 3.3.3 Escherichia coli

Articles studying vaccines for *E. coli* made up most articles in this review with a total of 43. The production system of 15 articles was described as beef herds or beef breed types, in 11 articles as dairy herds or dairy breed types, 1 article involved both beef and dairy cattle and 16 articles did not state the production type of cattle involved. Study sizes varied between a total of 10 and 4053 animals. Of the 43 articles on *E. coli* vaccines, 39 described vaccinating dams, while three reported on calf vaccination and one on vaccination of both calves and dams. Calves were challenged in 20 of the studies with either homologous (7 articles) or heterologous strains (5 articles) or had a combination of groups where some received homologous and some heterologous challenges (7 articles). In one of the articles, the challenge strain was not described. The results of 12 field trials involving commercial *E. coli* vaccines are described in Table 5, some of which also appear in Table 3 since they are combination vaccines with BRV/BCV. While 3 of the field trial articles showed advantageous results for incidence or treatment for calf diarrhea in vaccinated calves, 3 showed the opposite effect and 4 did not detect a difference between vaccinated and unvaccinated calves and 2 did not provide any statistics. One study that evaluated a combination vaccine for BRV and *E. coli* K99 did not evaluate the *E. coli* component as no natural challenge was evident and was only counted under BRV field studies for commercial vaccines (McNulty & Logan, 1987).

**Table 5:** Scoping review on vaccines for neonatal calf diarrhea: results of field trials for commercial *E. coli* vaccines with natural challenge

**Author, year**	**Vaccine name, vaccine type, pathogens, route, recipient**	**Study Group names (number in group)**	**Outcome**	**Result**
Krogh, 1983	Coligen, killed, 4-strain *E. coli*, route not stated, dam Vicogen, killed, K99 *E. coli*, route not stated, dam	1. Coligen (109)	Incidence of diarrhea in first week of life	Statistically lower in Vicogen group than placebo
		2. Vicogen (73)	Diarrhea-specific mortality	No statistics provided; mortality: Coligen group 1/109, Vicogen group 2/73, placebo group 2/114.
		3. Placebo (114)		
Schipper et al., 1984	Vicoten, killed, *E. coli*, route not stated, dam Coligen, killed, *E. coli*, route not stated, dam K99, killed, *E. coli*, route not stated, dam	1. Vicoten (1137)	Incidence of diarrhea	No difference between groups in year 1 of study; statistically lower in unvaccinated group in year 2 of study
		2. Coligen (754)		
		3. K99 (365)		
		4. Unvaccinated (948)		
Loucks et al., 1985	Vicogen, killed, *E. coli*, subcutaneous, dam	1. Vaccinated (51)	Diarrhea severity scores, treatments for diarrhea (major and minor), mortality	No difference between groups
		2. Unvaccinated (51)		
Sihvonen & Miettinen, 1985	Coligen, killed *E. coli*, intramuscular, dam	1. Vaccinated (45)	Incidence of diarrhea	No statistics provided; abnormal or diarrheic feces with K 99 *E. coli* in vaccinated (30/45) versus non-vaccinated (31/56).
		2. Non-vaccinated (56)		
Waltner-Toews et al., 1985*				
Kohara et al., 1997*				
Bendali et al., 1999	No specific vaccine named, *E. coli*, no route specified, dam	1. Vaccinated (777)	Incidence of diarrhea	Statistically higher in vaccinated
		2. Non-vaccinated (2303)		
Le Rousic, 2000*				
Perk et al., 2000*				
Younis et al., 2009	Scour Guard 3, modified live BRV, BCV, K99 bacterin, no route stated, dam	1. Vaccinated (no group size stated)	K99 infection in diarrheic calves	Statistically lower in vaccinated
		2. Non-vaccinated (no group size stated)		
Meganck et al., 2014*				
Gomes Rocha et al., 2017*				

*See Table 3

#### 3.3.4 Bovine coronavirus

There were 14 articles that evaluated vaccines for BCV of which thirteen included vaccines in combination with either BRV, BRV and *E. coli*, or BRV, *E. coli*, BVD, and bovine parvovirus. Study sizes for BCV vaccines varied between 6 and 5770. Dairy was the production system in 5 articles on BCV vaccines, 2 named beef as the production system, 1 conducted trials in both beef and dairy herds and 6 did not state the production system. The vaccine recipient was the dam in 10 of the articles, while 4 articles described vaccinating calves. Results for six field trials with commercial BCV vaccines are described in Table 3. Two studies in Table 3 that used vaccines for BCV tested for excretion of BRV only and were not counted as a field trial for commercial BCV vaccines (de Leeuw et al., 1980; Rocha et al., 2017). Of the 6 field trials that included BCV vaccines, 2 reported advantageous results for the vaccinated animals, 2 reported no difference, 1 found worse outcomes for vaccinated animals, and 1 did not report statistics.

#### 3.3.5 Other pathogens

Bovine Viral Diarrhea virus was the sole target pathogen in 6 articles. Four studies were performed in dairy herds, one in beef herds and one did not state the production system. Study sizes were small, ranging between 10 and 48 animals and in all studies, calves were the vaccine recipients and were challenged intranasally. Four of the studies used commercial vaccines versus 2 that tested experimental vaccines. One study focused mostly on the effect of added injection of trace minerals concurrently with vaccination but found no differences to calves that did not receive the injectable trace minerals with respect to clinical outcomes. However, all calves were protected from BVDV2 infection (Bittar et al., 2018). The study in beef herds compared three types of commercial vaccines and placebo given to early weaned calves (at 62 – 92 days of age, median age 72.2 days), which were subsequently challenged with BVD2. There were no clinical differences between the groups, which was thought to be due to protection from maternally derived antibodies in these calves (Chamorro et al., 2015).

Five articles studied vaccines for *Cryptosporidium*, all of which involved experimental vaccines. Three of the studies on *Cryptosporidium* vaccines targeted the dam, while two had calves vaccinated. In 4 articles, calves were challenged orally while 1 field study relied on natural infection. Vaccines used were recombinant P23 protein, killed oocysts, or whole oocysts. Four of the five studies showed favorable results in vaccinated calves with respect to duration of diarrhea and/or number of oocysts shed, however the one large field trial was unsuccessful in showing efficacy of the vaccine under investigation.

One article evaluated an experimental vaccine for *Giardia duodenalis* prepared from sonicated trophozoites (Uehlinger et al., 2007). The authors were unable to show efficacy in preventing giardiasis or reducing cyst shedding in a small trial with three vaccinated and three control calves that underwent an oral challenge of *G. duodenalis* cysts.

One article compared diarrhea scores and virus shedding post challenge depending on the route of vaccination in a trial for a norovirus vaccine in gnotobiotic calves, however no statistics were offered in this preliminary study with 15 calves in 7 different trial groups (Han et al., 2006).

One article studied a commercial vaccine that included bovine parvovirus besides BRV, BCV, *E. coli* and BVD (Kohara et al., 1997). Dams were the vaccine recipient in this trial. The authors could not detect an increase in virus neutralizing antibody titers in either dams or calves post vaccination and did not detect bovine parvovirus in any of the calves by examining fecal suspensions by electron microscopy.

## 4 Discussion

### 4.1 Available literature

While there are many review articles and book chapters on NCD as a disease complex and the various pathogens including the use and efficacy of vaccines, a Pubmed search on meta-analyses of the terms “scours” or “diarrhea” AND “calf” or “calves” resulted in no meta-analysis studies evaluating the efficacy of vaccines for the prevention of NCD. The present scoping review was therefore needed to summarize the available literature and to point out areas where meta-analyses will be useful or where further research is needed.

Unfortunately, the full text for 40 articles included in the 213 articles that passed the screening level was not available through the Carlson Health Library. The search for older articles that might have been retrievable on the shelves of partner libraries could have been impacted by the concurrent Covid-19 pandemic, which impaired in-person access to library buildings. Although criteria for the inclusion of references into the review listed only peer reviewed journals and conference abstracts of 500 words or more, 1 reference is from a book titled “Advances in Experimental Medicine and Biology” (Welter, 1998). Its contributions are peer-reviewed and therefore it was deemed appropriate to include the article.

### 4.2 General comments on vaccination for NCD

Calf diarrhea is often described as a management problem that should be addressed with improved hygiene and colostrum management (Tyler et al., 1999). Failure of transfer of passive immunity (Bragg et al., 2020) or inadequate transfer of passive immunity (Pearson et al., 2019) is prevalent in beef calves and have been associated with risk factors such as births requiring assistance, twin births, and heifer dams, and may be an important contributor to NCD in beef calves. Vaccination of calves or dams for the prevention of calf diarrhea is sometimes described as a band-aid for poor management. For example, one prospective cohort study in this review found that vaccination of dams for *E. coli* was associated with an increased risk for calf diarrhea. The authors hypothesized that those farms with high incidence of diarrhea try to eliminate the disease by vaccination (Bendali et al., 1999). As such, vaccination should not be regarded as the only component for the prevention of NCD as part of a herd health program but may be helpful to enhance other measures.

Vaccination of calves for neonatal diarrhea is commonly viewed as problematic depending on the pathogen, because disease often occurs early on in life, often within the first days, when the administration of vaccines may be less effective due to an immature immune system or the interference of maternal antibodies. It is therefore not surprising that a majority of studies investigated vaccination of pregnant cows to evaluate its effect on calf diarrhea-related outcomes. On the other hand, how successful vaccination of dams for the protection of calves from NCD is in the field depends on the transfer of passive immunity, which can vary with timing and amount of colostrum consumed by calves. Poor uptake of colostrum by calves may be part of an NCD herd problem so that dam vaccination is unlikely to be the only solution.

The randomized blinded placebo-controlled field trial is the reference standard to evaluate vaccine efficacy. We found a limited number of field trials of variable quality, based on whether randomization to study groups, blinding of researchers, or use of a placebo in the control group was mentioned, for vaccines for each of the major pathogens. An assessment of study quality is not part of a scoping review, therefore none of the available articles were excluded based on limitations of study design or analysis (Tricco et al., 2018). Moreover, USDA does not require the publication of efficacy data for vaccine licensure (Moon & Bunn, 1993), which limits the available information. A common explanation for why a given vaccine did not show efficacy during a field trial provided in the included articles, is a mismatch between outbreak and vaccine strain or the contribution of other non-target pathogens. Therefore, the use of appropriate diagnostics to identify an outbreak strain and careful selection of a vaccine appear to be important in the successful implementation of vaccines for the prevention of calf diarrhea.

### 4.3 Individual pathogens

#### 4.3.1 Bovine rotavirus

Bovine rotavirus is divided into eight groups or species A – H based on properties of an inner viral capsid protein V6, with group A being the most prevalent. Groups are further subdivided into strains via characterization of G (glycoprotein) and P (protease-sensitive) types (Matthijnssens et al., 2012). A relatively recent study concluded that even though strain prevalence fluctuates over space and time, a predominant genotype (G6P[5]) exists (Papp et al., 2013). Vaccine failure has been attributed to a mismatch between field and vaccine strains (Kohara et al., 1997; Rocha et al., 2017). Based on the number and outcomes of published field trials for commercial vaccines, a meta-analysis of the available data would be helpful to further characterize the usefulness of these vaccines for herds struggling with BRV infections.

#### 4.3.2 Salmonella

*Salmonella enterica* (abbreviated *Salmonella* from here on), a zoonotic agent, is divided into serogroups based on the O antigen and named by capital letters A, B, C, etc (Peek & Divers, 2018). *Salmonella* are further subdivided into serovars of which *Salmonella typhimurium*, and *Salmonella dublin* are most commonly isolated from clinical cases in cattle (Mizuno et al., 2008). *Salmonella dublin* is the host adapted serotype in cattle that can result in calf diarrhea and pneumonia outbreaks as well as subclinically infected carriers (Habing et al., 2011). Antimicrobial resistance to *Salmonella* is widespread, emphasizing the need for preventative measures as treatment options are limited (Peek & Divers, 2018).

Failure of killed *Salmonella* vaccines has been attributed to antigenic variety of *Salmonella* organisms as well as the inability of killed vaccines to stimulate a cell-mediated immune response, which is deemed important to combat *Salmonella* infections besides a humoral response (Curtiss et al., 1993; Habing et al., 2011; Johnson et al., 1985). *Salmonella* in live vaccines are attenuated through mutations in global regulatory networks, e.g. in the DNA adenine methylase (dam) (Mohler et al., 2008) or through aromatic-dependent mutants (aroˉ) (Mukkur et al., 1991; Smith et al., 1993). Fecal shedding of the vaccine strain and colonization of lymphoid tissue as well as side effects of vaccination from endotoxins are of concern when developing and using modified live *Salmonella* vaccines. A commercial vaccine targeting *Salmonella newport* is on the market in the U.S. but was not captured by our review as its effect on calf health has not been evaluated (Hermesch et al., 2008). Given the sparsity of available field trials for commercially available *Salmonella* vaccines and given that none showed efficacy of the respective vaccines, a meta-analysis of those data is not likely to provide useful information. Given the complexity of *Salmonella* serovars and immunogenicity of *Salmonella* infections as well as the risks associated with vaccination, a systematic review of *Salmonella* vaccines would be more helpful in guiding veterinarians and producers faced with NCD due to *Salmonella* infections.

#### 4.3.3 Escherichia coli

*Escherichia coli* is a commensal important member of the gut microbiota in cattle. However, multiple pathogenic types exist, which are classified based on cell wall (O), capsular (K), fimbrial (F) or flagellar (H) antigens. It is important to understand that pilus antigens were formerly classified as K antigens, but have recently been reclassified as F antigens to avoid confusion, e.g. K-99 is now F-5 (Peek & Divers, 2018). The enterotoxigenic *E. coli* (ETEC) F-5 and F-41 are most commonly associated with diarrhea in calves. Enterotoxigenic *E. coli* produce two virulence factors, namely adhesins, which include fimbriae, and heat-labile and heat-stable enterotoxins (Dubreuil et al., 2016). Colostral antibody cross-protection to different fimbrial antigens does not exist so that even calves with good transfer of passive immunity may be susceptible to ETEC with different F antigens. Vaccines must be matched to the outbreak F antigen to be effective.

Given the relative abundance of field trials with commercial vaccines targeting *E. coli,* a meta-analysis of the available studies would be helpful in further evaluating the usefulness of these vaccines under field conditions.

#### 4.3.4 Bovine coronavirus

Bovine coronavirus is part of the *Betacoronavirus* 1 species, which also includes human enteric coronavirus, equine coronavirus, canine respiratory coronavirus and others (Hodnik et al., 2020). Coronaviruses have been isolated from diarrheic calves and adult cattle (winter dysentery) as well as cattle with respiratory disease. Antigenic variation exists but seems unrelated to the different disease syndromes (Bidokhti et al., 2012; Hasoksuz et al., 1999; Tsunemitsu & Saif, 1995). Furthermore, cross-protection after exposure to BCV and reinfection with a BCV isolated from a different disease syndrome has been observed (Cho et al., 2001) leading to the hypothesis that clinical signs do not depend on specific strains but rather on host factors (Suzuki et al., 2020). As virtually all field trials summarized in this review are combination vaccines covering mostly BRV or BRV and *E. coli* in addition to BCV, a meta-analysis exploring the efficacy of these products should either combine all such vaccines or focus on BRV/BCV vaccines.

#### 4.3.5 Other pathogens

There are many studies that evaluate the use of vaccines for BVD. However, we specifically searched for those studies where diarrhea was among the outcomes analyzed. Bovine Viral Diarrhea virus is a virus with complex pathogenicity and many studies on vaccines for this pathogen are interested in outcomes such as bovine respiratory disease, reproductive failure in female cattle, persistently infected calves, or mucosal disease of calves. Even though vaccination for BVD should be part of all cattle operations, BVD is typically not considered as one of the common pathogens involved in NCD. A herd infected with BVD would likely see other disease manifestations such as abortions or poor-doing persistently infected calves that would point towards a problem with BVD. We included BVD vaccines for completion in this review but realize the limited scope of publications on BVD vaccines we were able to include based on our criteria.

*Cryptosporidiidae* are coccidial protozoa with *C. parvum* being the species of most clinical importance in causing diarrhea in preweaned calves (Peek & Divers, 2018; Thomson et al., 2017). Despite widespread occurrence of cryptosporidiosis, there are few treatment options. One of the available drugs for the prevention of diarrhea caused by *Cryptosporidium* is halofuginone lactate, which is, however, not approved for use in cattle everywhere. No commercial vaccines are currently marketed for the prevention of cryptosporidiosis in calves despite preliminary trials that have shown some promise for successful vaccination. Further research into the possibility of preventing cryptosporidiosis is desirable.

*Giardia duodenalis* is another protozoan organism that has been associated with calf diarrhea in dairy calves (O'Handley et al., 1999) and beef calves, although one study found no performance differences between infected and uninfected feedlot calves with regards to average daily gain, dry matter intake and feed efficiency (Ralston et al., 2003). *Giardia* infection appears to be ubiquitous and may play a role in exacerbating the effects of other pathogens or nutritional or environmental stressors (Olson et al., 2004). Its role in calf health is poorly described and further research may help better understand its contributions.

A number of other viruses have been identified in the feces of diarrheic calves, including calicivirus, torovirus, astrovirus, norovirus, and enterovirus, some of which have also been found in the feces of healthy calves complicating the interpretation of such findings (Gomez & Weese, 2017). We only found one article on a norovirus vaccine and one on a combination vaccine that included parvovirus antigen. The pathogenesis and epidemiology of many viruses are still poorly understood and research at the basic level is needed to better understand their importance and role in NCD.

## Conclusions

Vaccines for NCD have mainly focused on the pathogens BRV, BCV, *E. coli*, and *Salmonella*. Commercial products have been tested in field trials for these pathogens and shown variable efficacy. Hence, meta-analyses exploring efficacy of these vaccines in field trials would be useful. No commercial *Salmonella* vaccine has shown efficacy for the prevention of NCD in a published field trial. A systematic review of *Salmonella* vaccines describing the complex nature of different serovars and immunogenicity of *Salmonella* infection in calves is needed. For various other pathogens implicated in causing NCD, e.g. *Cryptosporidium*, available studies have not evolved beyond experimental stage trials and require further research.

## Ethical Statement

The research conducted in this manuscript meets the criteria for conduct, reporting, editing and publication of scholarly work in medical journals.

In particular, guidelines on authorship, disclosure of financial support, conflicts of interest, non-author contributions, duplicate submissions and prior publication have been followed.

No animal or human subjects have been involved in the research; therefore, no corresponding approvals of review boards are necessary.

The preferred reporting items for systematic review and meta-analysis protocols (PRISMA-P) as well as the PRISMA Extension for Scoping Review guidelines were used as references to conduct the review, further ensuring that relevant guidelines were followed.

## Funding

This work was supported by a grant from the California Department of Food and Agriculture, agreement number 18-0623-000-SG

## Declaration of interests

The authors declare that they have no known competing financial interests or personal relationships that could have appeared to influence the work reported in this paper.

## Appendix Supplementary materials

Image, application 1

Image, application 2
