# Supplementary material for: Vaccination for the Prevention of Neonatal Calf Diarrhea in Cow-Calf Operations: A Scoping Review
Source: Vet Anim Sci. 2022 Feb 19;15:100238. doi: 10.1016/j.vas.2022.100238 (PMC8866090; doi:10.1016/j.vas.2022.100238)
Supplement: Supplementary file 1 [file mmc1.docx]

Appendix A to article in Veterinary and Animal Science “Vaccination for the Prevention of Neonatal Calf Diarrhea in Cow-Calf Operations: A Scoping Review”

G. Maier, J. Breitenbuecher, J.P. Gomez, F. Samah, E. Fausak, M. Van Noord

Full electronic search strategies for Medline (PubMed Interface) , CAB Abstracts (CAB Direct interface), and Biosis (Web of Science interface)

**PubMed**

| **Search** | **Query** | **Items Found** |
| --- | --- | --- |
| #1 | (("Cattle"[Mesh] OR cattle[tiab] OR cow[tiab] OR cows[tiab] OR bos[tiab] OR bovine[tiab] OR bovines[tiab] OR bovinae[tiab] OR heifer[tiab] OR heifers[tiab] OR bullocks[tiab] OR oxen[tiab] OR steer[tiab] OR steers[tiab] OR Angus[tiab] OR Ayrshire[tiab] OR Boran[tiab] OR Brahman[tiab] OR Brangus[tiab] OR Braunvieh[tiab] OR Charolais[tiab] OR Fleckvieh[tiab] OR Friesian[tiab] OR Gelbvieh[tiab] OR Gir[tiab] OR Hereford[tiab] OR Holstein[tiab] OR Jersey[tiab] OR Limousin[tiab] OR Longhorn[tiab] OR Nellore[tiab] OR Ongole[tiab] OR Sahiwal[tiab] OR Sanga[tiab] OR Shorthorn[tiab] OR Simmental[tiab] OR Wagyu[tiab] OR beef[ti] OR herd[ti]) AND (calf[tiab] OR calves[tiab] OR calving[tiab])) OR ((calf[tiab] OR calves[tiab] OR calving[tiab]) AND ("Animals, Newborn"[Mesh] OR "Animals, Suckling"[Mesh] OR neonatal[tiab] OR newborn[tiab] OR newborns[tiab] OR "pre-wean"[tiab] OR prewean[tiab] OR "pre-weaning"[tiab] OR preweaning[tiab] OR "pre-weaned"[tiab] OR preweaned[tiab] OR nursing[tiab] OR suckling[tiab] OR "after birth"[tiab] OR "before weaning"[tiab] OR young[tiab])) OR (calf[ti] OR calf[ot] OR calves[ti] OR calves[ot]) | 48,504 |
| #2 | "Bovine Virus Diarrhea-Mucosal Disease"[Mesh] OR "Gastroenteritis/veterinary"[Mesh] OR "Diarrhea"[Mesh] OR diarrhea[tiab] OR diarrheic[tiab] OR diarrheal[tiab] OR diarrhoea[tiab] OR diarrhoeal[tiab] OR scour[tiab] OR scours[tiab] OR scouring[tiab] OR "fecal score"[tiab] OR "faecal score"[tiab] OR "watery feces"[tiab] OR "watery faeces"[tiab] | 122,664 |
| #3 | "Escherichia coli"[Mesh] OR "Escherichia coli"[tiab] OR "E coli"[tiab] OR "Escherichia coli Infections"[Mesh] OR "Salmonella"[Mesh] OR Salmonella[tiab] OR Salmonellosis[tiab] OR Salmonelloses[tiab] OR "Salmonella Infections"[Mesh] OR "Clostridium"[Mesh] OR clostridium[tiab] OR clostridiales[tiab] OR clostridiaceae[tiab] OR clostridia[tiab] OR "Clostridium Infections"[Mesh] OR "Shigella"[Mesh] OR shigella[tiab] OR shigellosis[tiab] OR "shiga bacillus"[tiab] OR "Dysentery, Bacillary"[Mesh] OR "Yersinia"[Mesh:NoExp] OR "Yersinia enterocolitica"[Mesh] OR yersinia[tiab] OR yersiniosis[tiab] OR yersinioses[tiab] OR "Yersinia Infections"[Mesh] OR "Coronavirus, Bovine"[Mesh] OR coronavirus[tiab] OR coronaviruses[tiab] OR "Coronaviridae"[Mesh:NoExp] OR "Torovirus"[Mesh] OR "Torovirus Infections"[Mesh] OR toroviridae[tiab] OR torovirus[tiab] OR toroviruses[tiab] OR “Breda Virus”[tiab] OR “Berne Virus”[tiab] OR "Rotavirus"[Mesh] OR rotavirus[tiab] OR rotaviruses[tiab] OR "Rotavirus Infections"[Mesh] OR "Caliciviridae"[Mesh:NoExp] OR nebovirus[tiab] OR Neboviruses[tiab] OR "Norovirus"[Mesh:NoExp] OR norovirus[tiab] OR Noroviruses[tiab] OR "Diarrhea Viruses, Bovine Viral"[Mesh] OR "Bovine Viral Diarrhea Virus"[tw] OR "Bovine Viral Diarrhea Viruses"[tiab] OR "Bovine Diarrhea Virus"[tiab] OR "Bovine Diarrhea Viruses"[tiab] OR "Bovine Pestivirus"[tiab] OR "Bovine Pestiviruses"[tiab] OR BVDV[tiab] OR "Cryptosporidium"[Mesh] OR cryptosporidium[tw] OR cryptosporidiums[tiab] OR "Cryptosporidiosis"[Mesh] OR cryptosporidiosis[tiab] OR cryptosporidioses[tiab] OR cryptosporidium[tiab] OR "Giardia"[Mesh] OR giardia[tw] OR giardias[tiab] OR lamblia[tiab] OR lamblias[tiab] OR lambliasis[tiab] OR lambliases[tiab] OR "Giardiasis"[Mesh] OR "fecal pathogens"[tiab] OR "fecal pathogen"[tiab] OR "faecal pathogens"[tiab] OR "faecal pathogen"[tiab] | 599,188 |
| #4 | #1 AND (#2 OR #3) | 6,678 |
| #5 | "Vaccines"[Mesh] OR vaccine[tiab] OR vaccines[tiab] OR "Immunization"[Mesh] OR vaccination[tiab] OR vaccinations[tiab] OR immunization[tiab] OR immunizations[tiab] OR injection[tiab] OR injected[tiab] OR injections[tiab] OR inoculation[tiab] OR inoculations[tiab] OR inoculate[tiab] OR inoculates[tiab] OR inoculated[tiab] OR ScourGuard[tiab] OR ScourBos[tiab] OR Bovilis[tiab] OR Rotavec[tiab] OR Ecolizer[tiab] | 1,190,152 |
| #6 | #4 AND #5 | 1,489 |
| #7 | ((calf[tiab] OR calves[tiab] OR calving[tiab]) AND "Diarrhea/prevention and control"[Mesh]) | 179 |
| #8 | #6 OR #7 | 1,582 |
| #9 | #8 AND English[lang] | 1,457 |
| #10 | #9 NOT ("Letter" [Publication Type] OR "Editorial" [Publication Type] OR "Case Reports" [Publication Type]) AND Filters: **Publication date from 1950/01/01** | 1,443 |

**CAB Abstracts**

| **Search** | **Search Term** | **Results** |
| --- | --- | --- |
| #1 | (od:("Bos") OR up:(bos)) AND (neonatal OR newborn OR newborns OR "pre-wean" OR prewean OR "pre-weaning" OR preweaning OR "pre-weaned" OR preweaned OR nursing OR suckling OR "after birth" OR "before weaning" OR young OR calf OR calves OR calving) | 121,297 |
| #2 | title:((cattle OR cow OR cows OR bos OR bovine OR bovines OR bovinae OR heifer OR heifers OR bullocks OR oxen OR steer OR steers OR Angus OR Ayrshire OR Boran OR Brahman OR Brangus OR Braunvieh OR Charolais OR Fleckvieh OR Friesian OR Gelbvieh OR Gir OR Hereford OR Holstein OR Jersey OR Limousin OR Longhorn OR Nellore OR Ongole OR Sahiwal OR Sanga OR Shorthorn OR Simmental OR Wagyu) ) OR ab:((cattle OR cow OR cows OR bos OR bovine OR bovines OR bovinae OR heifer OR heifers OR bullocks OR oxen OR steer OR steers OR Angus OR Ayrshire OR Boran OR Brahman OR Brangus OR Braunvieh OR Charolais OR Fleckvieh OR Friesian OR Gelbvieh OR Gir OR Hereford OR Holstein OR Jersey OR Limousin OR Longhorn OR Nellore OR Ongole OR Sahiwal OR Sanga OR Shorthorn OR Simmental OR Wagyu) ) | 545,153 |
| #3 | title:((neonatal OR newborn OR newborns OR "pre-wean" OR prewean OR "pre-weaning" OR preweaning OR "pre-weaned" OR preweaned OR nursing OR suckling OR "after birth" OR "before weaning" OR young OR calf OR calves OR calving)) OR ab:((neonatal OR newborn OR newborns OR "pre-wean" OR prewean OR "pre-weaning" OR preweaning OR "pre-weaned" OR preweaned OR nursing OR suckling OR "after birth" OR "before weaning" OR young OR calf OR calves OR calving)) | 505,311 |
| #4 | #2 AND #3 | 98,302 |
| #5 | #1 OR #4 | 129,504 |
| #6 | diarrhea OR diarrheic OR diarrheal OR diarrhoea OR diarrhoeal OR scour OR scours OR scouring OR "fecal score" OR "faecal score" OR "watery feces" OR "watery faeces" | 86,879 |
| #7 | #5 AND #6 | 10,571 |
| #8 | "Escherichia coli" OR "E coli" OR Salmonella OR Salmonellosis OR Salmonelloses OR clostridium OR clostridiales OR clostridiaceae OR clostridia OR shigella OR shigellosis OR "shiga bacillus" OR yersinia OR yersiniosis OR yersinioses OR coronavirus OR coronaviruses OR toroviridae OR torovirus OR toroviruses OR "Breda Virus" OR "Berne Virus" OR rotavirus OR rotaviruses OR nebovirus OR Neboviruses OR norovirus OR Noroviruses OR "Bovine Viral Diarrhea Virus" OR "Bovine Viral Diarrhea Viruses" OR "Bovine Diarrhea Virus" OR "Bovine Diarrhea Viruses" OR "Bovine Pestivirus" OR "Bovine Pestiviruses" OR BVDV OR cryptosporidium OR cryptosporidiums OR cryptosporidiosis OR cryptosporidioses OR cryptosporidium OR giardia OR giardias OR lamblia OR lamblias OR lambliasis OR lambliases OR giardiasis OR "fecal pathogens" OR "fecal pathogen" OR "faecal pathogens" OR "faecal pathogen" | 298,904 |
| #9 | #5 AND #8 | 11,628 |
| #10 | #7 OR #9 | 15,948 |
| #11 | vaccine OR vaccines OR vaccination OR vaccinations OR immunization OR immunizations OR inoculation OR inoculations OR inoculate OR inoculates OR inoculated OR ScourGuard OR ScourBos OR Bovilis OR Rotavec OR Ecolizer | 428,505 |
| #12 | #10 AND #11 AND yr:[1950 TO 2019] AND Language: English | 2,209 |
| #13 | #12 AND Document type: Journal article OR Journal issue OR Conference proceedings OR Conference paper OR Miscellaneous OR Abstract only | 2,044 |

**BIOSIS**

| **Search** | **Query** | **Items Found** |
| --- | --- | --- |
| #1 | TI=(cattle OR cow OR cows OR bos OR bovine OR bovines OR bovinae OR heifer OR heifers OR bullocks OR oxen OR steer OR steers OR Angus OR Ayrshire OR Boran OR Brahman OR Brangus OR Braunvieh OR Charolais OR Fleckvieh OR Friesian OR Gelbvieh OR Gir OR Hereford OR Holstein OR Jersey OR Limousin OR Longhorn OR Nellore OR Ongole OR Sahiwal OR Sanga OR Shorthorn OR Simmental OR Wagyu) OR TS=(cattle OR cow OR cows OR bos OR bovine OR bovines OR bovinae OR heifer OR heifers OR bullocks OR oxen OR steer OR steers OR Angus OR Ayrshire OR Boran OR Brahman OR Brangus OR Braunvieh OR Charolais OR Fleckvieh OR Friesian OR Gelbvieh OR Gir OR Hereford OR Holstein OR Jersey OR Limousin OR Longhorn OR Nellore OR Ongole OR Sahiwal OR Sanga OR Shorthorn OR Simmental OR Wagyu) | 591,045 |
| #2 | TI=(neonatal OR newborn OR newborns OR "pre-wean" OR prewean OR "pre-weaning" OR preweaning OR "pre-weaned" OR preweaned OR nursing OR suckling OR "after birth" OR "before weaning" OR young OR calf OR calves OR calving) OR TS=(neonatal OR newborn OR newborns OR "pre-wean" OR prewean OR "pre-weaning" OR preweaning OR "pre-weaned" OR preweaned OR nursing OR suckling OR "after birth" OR "before weaning" OR young OR calf OR calves OR calving) | 991,373 |
| #3 | #1 AND #2 | 67,252 |
| #4 | TI=(diarrhea OR diarrheic OR diarrheal OR diarrhoea OR diarrhoeal OR scour OR scours OR scouring OR "fecal score" OR "faecal score" OR "watery feces" OR "watery faeces" OR "Escherichia coli" OR "E coli" OR Salmonella OR Salmonellosis OR Salmonelloses OR clostridium OR clostridiales OR clostridiaceae OR clostridia OR shigella OR shigellosis OR "shiga bacillus" OR yersinia OR yersiniosis OR yersinioses OR coronavirus OR coronaviruses OR toroviridae OR torovirus OR toroviruses OR "Breda Virus" OR "Berne Virus" OR rotavirus OR rotaviruses OR nebovirus OR Neboviruses OR norovirus OR Noroviruses OR "Bovine Viral Diarrhea Virus" OR "Bovine Viral Diarrhea Viruses" OR "Bovine Diarrhea Virus" OR "Bovine Diarrhea Viruses" OR "Bovine Pestivirus" OR "Bovine Pestiviruses" OR BVDV OR cryptosporidium OR cryptosporidiums OR cryptosporidiosis OR cryptosporidioses OR cryptosporidium OR giardia OR giardias OR lamblia OR lamblias OR lambliasis OR lambliases OR giardiasis OR "fecal pathogens" OR "fecal pathogen" OR "faecal pathogens" OR "faecal pathogen") OR TS=( diarrhea OR diarrheic OR diarrheal OR diarrhoea OR diarrhoeal OR scour OR scours OR scouring OR "fecal score" OR "faecal score" OR "watery feces" OR "watery faeces" OR "Escherichia coli" OR "E coli" OR Salmonella OR Salmonellosis OR Salmonelloses OR clostridium OR clostridiales OR clostridiaceae OR clostridia OR shigella OR shigellosis OR "shiga bacillus" OR yersinia OR yersiniosis OR yersinioses OR coronavirus OR coronaviruses OR toroviridae OR torovirus OR toroviruses OR "Breda Virus" OR "Berne Virus" OR rotavirus OR rotaviruses OR nebovirus OR Neboviruses OR norovirus OR Noroviruses OR "Bovine Viral Diarrhea Virus" OR "Bovine Viral Diarrhea Viruses" OR "Bovine Diarrhea Virus" OR "Bovine Diarrhea Viruses" OR "Bovine Pestivirus" OR "Bovine Pestiviruses" OR BVDV OR cryptosporidium OR cryptosporidiums OR cryptosporidiosis OR cryptosporidioses OR cryptosporidium OR giardia OR giardias OR lamblia OR lamblias OR lambliasis OR lambliases OR giardiasis OR "fecal pathogens" OR "fecal pathogen" OR "faecal pathogens" OR "faecal pathogen") | 772,099 |
| #5 | #3 AND #4 | 6,433 |
| #6 | TI=(vaccine OR vaccines OR vaccination OR vaccinations OR immunization OR immunizations OR inoculation OR inoculations OR inoculate OR inoculates OR inoculated OR ScourGuard OR ScourBos OR Bovilis OR Rotavec OR Ecolizer) OR TS=(vaccine OR vaccines OR vaccination OR vaccinations OR immunization OR immunizations OR inoculation OR inoculations OR inoculate OR inoculates OR inoculated OR ScourGuard OR ScourBos OR Bovilis OR Rotavec OR Ecolizer) | 936,885 |
| #7 | #5 AND #6 | 1,489 |
| #8 | #7 *AND* **LANGUAGE:** (English)  Indexes=BIOSIS Previews Timespan=1950-2019 | 1,333 |
