# Supplementary material for: Vaccination for the Prevention of Neonatal Calf Diarrhea in Cow-Calf Operations: A Scoping Review
Source: Vet Anim Sci. 2022 Feb 19;15:100238. doi: 10.1016/j.vas.2022.100238 (PMC8866090; doi:10.1016/j.vas.2022.100238)
Supplement: Supplementary file 2 [file mmc2.docx]

Appendix B to article in Veterinary and Animal Science “Vaccination for the Prevention of Neonatal Calf Diarrhea in Cow-Calf Operations: A Scoping Review”

G. Maier, J. Breitenbuecher, J.P. Gomez, F. Samah, E. Fausak, M. Van Noord

References included in scoping review by target pathogen, in chronological order. Categories are not mutually exclusive such that a reference may appear more than once.

**Rotavirus vaccine studies**

Acres, S. D., & Radostits, O. M. (1976). The efficacy of a modified live reo-like virus vaccine and an E. coli bacterin for prevention of acute undifferentiated neonatal diarrhea of beef calves. *Can Vet J, 17*(8), 197-212. https://www.ncbi.nlm.nih.gov/pmc/articles/PMC1697302/pdf/canvetj00393-0005.pdf

Thurber, E. T., Bass, E. P., & Beckenhauer, W. H. (1977). Field trial evaluation of a reo-coronavirus calf diarrhea vaccine. *Can J Comp Med, 41*(2), 131-136.

Woode, G. N., Bew, M. E., & Dennis, M. J. (1978). Studies on cross protection induced in calves by rotaviruses of calves, children and foals. *Vet Rec, 103*(2), 32-34. https://doi.org/10.1136/vr.103.2.32.

de Leeuw, P. W., Ellens, D. J., Talmon, F. P., Zimmer, G. N., & Kommerij, R. (1980). Rotavirus infections in calves: efficacy of oral vaccination in endemically infected herds. *Res Vet Sci, 29*(2), 142-147.

Snodgrass, D. R., Fahey, K. J., Wells, P. W., Campbell, I., & Whitelaw, A. (1980). Passive immunity in calf rotavirus infections: maternal vaccination increases and prolongs immunoglobulin G1 antibody secretion in milk. *Infect Immun, 28*(2), 344-349.

Hudson, D. (1981). Rota-coronavirus vaccination of pregnant cows. *Modern Veterinary Practice, 62*(8), 626-628. https://www.cabdirect.org/cabdirect/abstract/19812285929

Eichhorn, W., Bachmann, P. A., Baljer, G., Plank, P., & Schneider, P. (1983). Vaccination of cows with a combined rotavirus/enterotoxigenic "E. coli" K99 vaccine to protect newborn calves against diarrhoea. *Dev Biol Stand, 53*, 237-243.

Snodgrass, D. R., Nagy, L. K., Sherwood, D., & Campbell, I. (1982). Passive immunity in calf diarrhea: vaccination with K99 antigen of enterotoxigenic Escherichia coli and rotavirus. Infect Immun, 37(2), 586-591.

Burki, F., Schusser, G., & Szekely, H. (1983). Clinical, virological and serological evaluation of the efficacy of peroral live rotavirus vaccination in calves kept under normal husbandry conditions. *Zentralbl Veterinarmed B, 30*(4), 237-250. https://doi.org/10.1111/j.1439-0450.1983.tb01839.x.

Saif, L. J., Redman, D. R., Smith, K. L., & Theil, K. W. (1983). Passive immunity to bovine rotavirus in newborn calves fed colostrum supplements from immunized or nonimmunized cows. *Infect Immun, 41*(3), 1118-1131.

Castrucci, G., Frigeri, F., Ferrari, M., Cilli, V., Caleffi, F., Aldrovandi, V., & Nigrelli, A. (1984a). The efficacy of colostrum from cows vaccinated with rotavirus in protecting calves to experimentally induced rotavirus infection. *Comp Immunol Microbiol Infect Dis, 7*(1), 11-18. <https://doi.org/10.1016/0147-9571(84)90011-0>.

Castrucci, G., Frigeri, F., Ferrari, M., Cilli, V., Caleffi, F., Aldrovandi, V., & Nigrelli, A. (1984b). Studies on passive immunity in calf rotaviral infections. *Proceedings of the 13th World Congress on Diseases of Cattle, Durban, South Africa, 1*, 341-346. https://www.cabdirect.org/cabdirect/abstract/19852255203.

de Leeuw, P. W., & Tiessink, J. W. (1985). Laboratory experiments on oral vaccination of calves against rotavirus or coronavirus induced diarrhoea. *Zentralbl Veterinarmed B, 32*(1), 55-64. <https://doi.org/10.1111/j.1439-0450.1985.tb01937.x>.

Waltner-Toews, D., Martin, S. W., Meek, A. H., McMillan, I., & Crouch, C. F. (1985). A field trial to evaluate the efficacy of a combined rotavirus-coronavirus/Escherichia coli vaccine in dairy cattle. *Can J Comp Med, 49*(1), 1-9.

Burki, F., Mostl, K., Spiegl, E., Horvath, E., & Szekely, H. (1986). Reduction of rotavirus-, coronavirus- and E. coli-associated calf-diarrheas in a large-size dairy herd by means of dam vaccination with a triple-vaccine. *Zentralbl Veterinarmed B, 33*(4), 241-252. https://doi.org/10.1111/j.1439-0450.1986.tb00029.x.

Snodgrass, D. R., Stewart, J., Taylor, J., Krautil, F. L., & Smith, M. L. (1982). Diarrhoea in dairy calves reduced by feeding colostrum from cows vaccinated with rotavirus. *Res Vet Sci, 32*(1), 70-73.

Snodgrass, D. R. (1986). Evaluation of a combined rotavirus and enterotoxigenic Escherichia coli vaccine in cattle. *Vet Rec, 119*(2), 39-42. https://doi.org/10.1136/vr.119.2.39.

Castrucci, G., Frigeri, F., Angelillo, V., Ferrari, M., Cilli, V., & Aldrovandi, V. (1987). Field trial evaluation of an inactivated rotavirus vaccine against neonatal diarrhea of calves. *Eur J Epidemiol, 3*(1), 5-9. https://doi.org/10.1007/bf00145064.

McNulty, M. S., & Logan, E. F. (1987). Effect of vaccination of the dam on rotavirus infection in young calves. *Vet Rec, 120*(11), 250-252. https://doi.org/10.1136/vr.120.11.250.

Woode, G. N., Zheng, S. L., Rosen, B. I., Knight, N., Gourley, N. E., & Ramig, R. F. (1987). Protection between different serotypes of bovine rotavirus in gnotobiotic calves: specificity of serum antibody and coproantibody responses. *J Clin Microbiol, 25*(6), 1052-1058.

Archambault, D., Morin, G., Elazhary, Y., Roy, R. S., & Joncas, J. H. (1988). Immune response of pregnant heifers and cows to bovine rotavirus inoculation and passive protection to rotavirus infection in newborn calves fed colostral antibodies or colostral lymphocytes. *Am J Vet Res, 49*(7), 1084-1091.

Castrucci, G., Frigeri, F., Ferrari, M., Aldrovandi, V., Tassini, F., & Gatti, R. (1988). The protection of newborn calves against experimental rotavirus infection by feeding mammary secretions from vaccinated cows. *Microbiologica, 11*(4), 379-385.

Mostl, K., & Burki, F. (1988). Incidence of diarrhoea and of rotavirus- and coronavirus-shedding in calves, whose dams had been vaccinated with an experimental oil-adjuvanted vaccine containing bovine rotavirus and bovine coronavirus. *Zentralbl Veterinarmed B, 35*(3), 186-196. https://doi.org/10.1111/j.1439-0450.1988.tb00486.x.

Tsunemitsu, H., Shimizu, M., Hirai, T., Yonemichi, H., Kudo, T., Mori, K., & Onoe, S. (1989). Protection against bovine rotaviruses in newborn calves by continuous feeding of immune colostrum. *Nihon Juigaku Zasshi, 51*(2), 300-308. <https://doi.org/10.1292/jvms1939.51.300>.

Bellinzoni, R. C., Blackhall, J., Baro, N., Auza, N., Mattion, N., Casaro, A., La Torre, J. L., & Scodeller, E. A. (1989). Efficacy of an inactivated oil-adjuvanted rotavirus vaccine in the control of calf diarrhoea in beef herds in Argentina. *Vaccine, 7*(3), 263-268. https://doi.org/10.1016/0264-410x(89)90241-7.

Castrucci, G., Frigeri, F., Ferrari, M., Aldrovandi, V., Angelillo, V., & Gatti, R. (1989). Immunization against bovine rotaviral infection. *Eur J Epidemiol, 5*(3), 279-284. https://doi.org/10.1007/bf00144827.

Castrucci, G., Frigeri, F., Ferrari, M., Aldrovandi, V., & Tassini, F. (1989). Further studies on passive immunization of newborn calves against rotaviral infection. *Comp Immunol Microbiol Infect Dis, 12*(3), 71-76. https://doi.org/10.1016/0147-9571(89)90051-9.

Cornaglia, E. M., Fernandez, F. M., Gottschalk, M., Barrandeguy, M. E., Luchelli, A., Pasini, M. I., Saif, L. J., Parraud, J. R., Romat, A., & Schudel, A. A. (1992). Reduction in morbidity due to diarrhea in nursing beef calves by use of an inactivated oil-adjuvanted rotavirus-Escherichia coli vaccine in the dam. *Vet Microbiol, 30*(2-3), 191-202. <https://doi.org/10.1016/0378-1135(92)90113-8>.

Castrucci, G., Ferrari, M., Angelillo, V., Rigonat, F., & Capodicasa, L. (1993). Field evaluation of the efficacy of Romovac 50, a new inactivated, adjuvanted bovine rotavirus vaccine. *Comp Immunol Microbiol Infect Dis, 16*(3), 235-239. https://doi.org/10.1016/0147-9571(93)90150-4.

Chung, C., Chang, C., Cho, J., Park, B., Cho, I., An, S., Lee, H., Lym, Y., Lee, K., & Kim, J. (1996). Immunogenicity and efficacy of an attenuated bovine rotavirus-coronavirus experimental vaccine. *RDA Journal of Agricultural Science, Veterinary, 38*(2), 721-728. https://www.cabdirect.org/cabdirect/abstract/19972208900

Kohara, J., Hirai, T., Mori, K., Ishizaki, H., & Tsunemitsu, H. (1997). Enhancement of passive immunity with maternal vaccine against newborn calf diarrhea. *J Vet Med Sci, 59*(11), 1023-1025. https://doi.org/10.1292/jvms.59.1023.

Fernandez, F. M., Conner, M. E., Hodgins, D. C., Parwani, A. V., Nielsen, P. R., Crawford, S. E., Estes, M. K., & Saif, L. J. (1998). Passive immunity to bovine rotavirus in newborn calves fed colostrum supplements from cows immunized with recombinant SA11 rotavirus core-like particle (CLP) or virus-like particle (VLP) vaccines. *Vaccine, 16*(5), 507-516. https://doi.org/10.1016/s0264-410x(97)80004-7.

Le Rousic, S., Klein, N., Houghton, S., & Charleston, B. (2000). Use of colostrum from rotavirus-immunised cows as a single feed to prevent rotavirus-induced diarrhoea in calves. *Vet Rec, 147*(6), 160-161. https://doi.org/10.1136/vr.147.6.160.

Perk, K., Moussa, A. A., Tromp, A. M., Reda, I. M., Refai, M., Friedman, A., Farid, A. F., Gallily, O., Salah, S. M., & Saif, L. (2000). Neonatal diarrheal disease of dairy cattle in Egypt and Israel. *Israel Journal of Veterinary Medicine, 55*(1), 13-18. https://www.cabdirect.org/cabdirect/abstract/20002216311

Jayappa, H., Davis, R., Dierks, L., Sweeney, D., & Wasmoen, T. (2008). Demonstration of passive protection in neonatal calves against colibacillosis following immunization of pregnant heifers at 3 months of gestation. *Vet Ther, 9*(4), 283-289.

Gonzalez, D. D., Mozgovoj, M. V., Bellido, D., Rodriguez, D. V., Fernandez, F. M., Wigdorovitz, A., Parreno, V. G., & Dus Santos, M. J. (2010). Evaluation of a bovine rotavirus VP6 vaccine efficacy in the calf model of infection and disease. *Vet Immunol Immunopathol, 137*(1-2), 155-160. https://doi.org/10.1016/j.vetimm.2010.04.015. Epub 2010 Apr 29.

Meganck, V., Hoflack, G., Piepers, S., & Opsomer, G. (2015). Evaluation of a protocol to reduce the incidence of neonatal calf diarrhoea on dairy herds. *Prev Vet Med, 118*(1), 64-70. https://doi.org/10.1016/j.prevetmed.2014.11.007. Epub 2014 Nov 15.

Rocha, T. G., Silva, F. D., Gregori, F., Alfieri, A. A., Buzinaro, M. D., & Fagliari, J. J. (2017). Longitudinal study of bovine rotavirus group A in newborn calves from vaccinated and unvaccinated dairy herds. *Trop Anim Health Prod, 49*(4), 783-790. https://doi.org/10.1007/s11250-017-1263-2. Epub 2017 Mar 20.

**Coronavirus vaccine studies**

Thurber, E. T., Bass, E. P., & Beckenhauer, W. H. (1977). Field trial evaluation of a reo-coronavirus calf diarrhea vaccine. *Can J Comp Med, 41*(2), 131-136.

Hudson, D. (1981). Rota-coronavirus vaccination of pregnant cows. *Modern Veterinary Practice, 62*(8), 626-628. https://www.cabdirect.org/cabdirect/abstract/19812285929

Burki, F., Schusser, G., & Szekely, H. (1983). Clinical, virological and serological evaluation of the efficacy of peroral live rotavirus vaccination in calves kept under normal husbandry conditions. *Zentralbl Veterinarmed B, 30*(4), 237-250. https://doi.org/10.1111/j.1439-0450.1983.tb01839.x.

de Leeuw, P. W., & Tiessink, J. W. (1985). Laboratory experiments on oral vaccination of calves against rotavirus or coronavirus induced diarrhoea. *Zentralbl Veterinarmed B, 32*(1), 55-64. <https://doi.org/10.1111/j.1439-0450.1985.tb01937.x>.

Waltner-Toews, D., Martin, S. W., Meek, A. H., McMillan, I., & Crouch, C. F. (1985). A field trial to evaluate the efficacy of a combined rotavirus-coronavirus/Escherichia coli vaccine in dairy cattle. *Can J Comp Med, 49*(1), 1-9.

Burki, F., Mostl, K., Spiegl, E., Horvath, E., & Szekely, H. (1986). Reduction of rotavirus-, coronavirus- and E. coli-associated calf-diarrheas in a large-size dairy herd by means of dam vaccination with a triple-vaccine. *Zentralbl Veterinarmed B, 33*(4), 241-252. https://doi.org/10.1111/j.1439-0450.1986.tb00029.x.

Mostl, K., & Burki, F. (1988). Incidence of diarrhoea and of rotavirus- and coronavirus-shedding in calves, whose dams had been vaccinated with an experimental oil-adjuvanted vaccine containing bovine rotavirus and bovine coronavirus. *Zentralbl Veterinarmed B, 35*(3), 186-196. https://doi.org/10.1111/j.1439-0450.1988.tb00486.x.

Chung, C., Chang, C., Cho, J., Park, B., Cho, I., An, S., Lee, H., Lym, Y., Lee, K., & Kim, J. (1996). Immunogenicity and efficacy of an attenuated bovine rotavirus-coronavirus experimental vaccine. *RDA Journal of Agricultural Science, Veterinary, 38*(2), 721-728. https://www.cabdirect.org/cabdirect/abstract/19972208900

Kohara, J., Hirai, T., Mori, K., Ishizaki, H., & Tsunemitsu, H. (1997). Enhancement of passive immunity with maternal vaccine against newborn calf diarrhea. *J Vet Med Sci, 59*(11), 1023-1025. https://doi.org/10.1292/jvms.59.1023.

Welter, M. W. (1998). Adaptation and serial passage of bovine coronavirus in an established diploid swine testicular cell line and subsequent development of a modified live vaccine. *Adv Exp Med Biol, 440*, 707-711. https://doi.org/10.1007/978-1-4615-5331-1_91.

Le Rousic, S., Klein, N., Houghton, S., & Charleston, B. (2000). Use of colostrum from rotavirus-immunised cows as a single feed to prevent rotavirus-induced diarrhoea in calves. *Vet Rec, 147*(6), 160-161. https://doi.org/10.1136/vr.147.6.160.

Jayappa, H., Davis, R., Dierks, L., Sweeney, D., & Wasmoen, T. (2008). Demonstration of passive protection in neonatal calves against colibacillosis following immunization of pregnant heifers at 3 months of gestation. *Vet Ther, 9*(4), 283-289.

Meganck, V., Hoflack, G., Piepers, S., & Opsomer, G. (2015). Evaluation of a protocol to reduce the incidence of neonatal calf diarrhoea on dairy herds. *Prev Vet Med, 118*(1), 64-70. https://doi.org/10.1016/j.prevetmed.2014.11.007. Epub 2014 Nov 15.

Rocha, T. G., Silva, F. D., Gregori, F., Alfieri, A. A., Buzinaro, M. D., & Fagliari, J. J. (2017). Longitudinal study of bovine rotavirus group A in newborn calves from vaccinated and unvaccinated dairy herds. *Trop Anim Health Prod, 49*(4), 783-790. https://doi.org/10.1007/s11250-017-1263-2. Epub 2017 Mar 20.

***E. coli* vaccine studies**

Sellers, K. C., Smith, H. W., & Pook, H. L. (1962). The evaluation of a dead Escherichia coli vaccine administered during pregnancy in the prevention of scouring (diarrhoea) in calves. *Veterinary Record, 74*, 203-204. https://www.cabdirect.org/cabdirect/abstract/19622202169

Gay, C. C., McKay, K. A., & Barnum, D. A. (1964a). Studies on colibacillosis of calves. II. A clinical evaluation of the efficacy of vaccination of the dam as a means of preventing colibacillosis of the calf. *Canadian Veterinary Journal, 5*, 297-308. https://www.cabdirect.org/cabdirect/abstract/19652202431

Gay, C. C., McKay, K. A., & Barnum, D. A. (1964b). Studies on colibacillosis of calves. III. The experimental reproduction of colibacillosis. *Canadian Veterinary Journal, 5*, 314-325. https://www.cabdirect.org/cabdirect/abstract/19652204963

Myers, L. L., Newman, F. S., Wilson, R. A., & Catlin, J. E. (1973). Passive immunization of calves against experimentally induced enteric colibacillosis by vaccination of dams. *Am J Vet Res, 34*(1), 29-33.

Newman, F. S., Myers, L. L., Firehammer, B. D., & Catlin, J. E. (1973). Prevention of experimentally induced enteric colibacillosis in newborn calves. *Infect Immun, 8*(4), 540-543.

Varga, J., & Farid, A. F. (1975). Vaccination experiments on prevention of E. coli diarrhoea in suckling calves. I. Use of aluminium gel adjuvant. *Acta Vet Acad Sci Hung, 25*(2-3), 153-161.

Varga, J., & Farid, A. F. (1976). Vaccination experiments on prevention of E. coli-diarrhoea in suckling calves. II. Use of vaccines containing Freund adjuvant. *Acta Vet Acad Sci Hung, 26*(1), 49-54.

Acres, S. D., & Radostits, O. M. (1976). The efficacy of a modified live reo-like virus vaccine and an E. coli bacterin for prevention of acute undifferentiated neonatal diarrhea of beef calves. *Can Vet J, 17*(8), 197-212. https://www.ncbi.nlm.nih.gov/pmc/articles/PMC1697302/pdf/canvetj00393-0005.pdf

Myers, L. L. (1976). Vaccination of cows with an Escherichia coli bacterin for the prevention of naturally occurring diarrheal disease in their calves. *Am J Vet Res, 37*(7), 831-834.

Wilson, R. A., & Jutila, J. W. (1976). Experimental neonatal colibacillosis in cows: immunoglobin classes involved in protection. *Infect Immun, 13*(1), 100-107.

Acres, S. D., Isaacson, R. E., Babiuk, L. A., & Kapitany, R. A. (1979). Immunization of calves against enterotoxigenic colibacillosis by vaccinating dams with purified K99 antigen and whole cell bacterins. *Infect Immun, 25*(1), 121-126.

Contrepois, M., Girardeau, J. P., Dubourguier, H. C., Gouet, P., & Levieux, D. (1978). Specific protection by colostrum from cows vaccinated with the K 99 antigen in newborn calves experimentally infected with E. coli Ent+ K99+. *Ann Rech Vet, 9*(2), 385-388.

Bagley, C. V., & Call, J. W. (1979). Vaccination of the dam by the intramuscular or deep subcutaneous route to prevent neonatal calf enteric colibacillosis. *Am J Vet Res, 40*(9), 1285-1287.

Myers, L. L. (1979). Passive protection of calves against enteric colibacillosis by vaccination of their dams with capsular and fimbrial K antigens of Escherichia coli. 427-437. https://www.cabdirect.org/cabdirect/abstract/19792240506

Acosta-Martinez, F., Gyles, C. L., & Butler, D. G. (1980). Escherichia coli heat-stable enterotoxin in feces and intestines of calves with diarrhea. *Am J Vet Res, 41*(7), 1143-1149.

Myers, L. L. (1980). Passive protection of calves against experimentally induced and naturally occurring enteric colibacillosis. *Am J Vet Res, 41*(12), 1952-1956.

Nagy, B. (1980). Vaccination of cows with a K99 extract to protect newborn calves against experimental enterotoxic colibacillosis. *Infect Immun, 27*(1), 21-24.

Kornitzer, I., Tamarin, R., Brenner, Y., Cohen, A., Trainin, Z., & Davidson, M. (1980). Protection of calves against experimental enterotoxigenic Escherichia coli infection by immunization of dams. Refuah Veterinarith, 37(3), 71-80. https://www.cabdirect.org/cabdirect/abstract/19812286547

Eichhorn, W., Bachmann, P. A., Baljer, G., Plank, P., & Schneider, P. (1983). Vaccination of cows with a combined rotavirus/enterotoxigenic "E. coli" K99 vaccine to protect newborn calves against diarrhoea. *Dev Biol Stand, 53*, 237-243.

Snodgrass, D. R., Stewart, J., Taylor, J., Krautil, F. L., & Smith, M. L. (1982). Diarrhoea in dairy calves reduced by feeding colostrum from cows vaccinated with rotavirus. *Res Vet Sci, 32*(1), 70-73.

Krogh, H. V. (1983). Infection with enterotoxigenic Escherichia coli in calves and protection of the calves by vaccination of the dams. *Ann Rech Vet, 14*(4), 522-525.

Schipper, I. A., Pommer, J., Landblom, D., Danielson, R., & Slanger, W. (1984). A two-year study of vaccination in the prevention of bovine E. coli diarrhea. *North Dakota Farm Research, 41*(5), 23-29. https://www.cabdirect.org/cabdirect/abstract/19842245365

Contrepois, M. G., & Girardeau, J. P. (1985). Additive protective effects of colostral antipili antibodies in calves experimentally infected with enterotoxigenic Escherichia coli. *Infect Immun, 50*(3), 947-949. https://iai.asm.org/content/iai/50/3/947.full.pdf

Loucks, M. E., Morrill, J. L., & Dayton, A. D. (1985). Effect of prepartum vaccination with K99 Escherichia coli vaccine on maternal and calf blood antibody concentration and calf health. *J Dairy Sci, 68*(7), 1841-1847. https://doi.org/10.3168/jds.S0022-0302(85)81037-7.

Sihvonen, L., & Miettinen, P. (1985). Rotavirus and Enterotoxigenic Escherichia-Coli Infections of Calves on a Closed Finnish Dairy Farm. *Acta Veterinaria Scandinavica, 26*(2), 205-217.

Waltner-Toews, D., Martin, S. W., Meek, A. H., McMillan, I., & Crouch, C. F. (1985). A field trial to evaluate the efficacy of a combined rotavirus-coronavirus/Escherichia coli vaccine in dairy cattle. *Can J Comp Med, 49*(1), 1-9.

Collins, N. F., Halbur, T., Schwenck, W. H., Hoogeveen, P., Pierce, R. L., Behan, R. W., & Pankratz, D. (1988). Duration of immunity and efficacy of an oil emulsion Escherichia coli bacterin in cattle. *Am J Vet Res, 49*(5), 674-677.

Valente, C., Fruganti, G., Tesei, B., Ciorba, A., Cardaras, P., Floris, A., & Bordoni, E. (1988). Vaccination of pregnant cows with K99 antigen of enterotoxigenic Escherichia coli and protection by colostrum in newborn calves. *Comp Immunol Microbiol Infect Dis, 11*(3-4), 189-198. https://doi.org/10.1016/0147-9571(88)90037-9.

Acres, S. D., Isaacson, R. E., Khachatourians, G., Babiuk, L., & Kapitany, R. A. (1979). Vaccination of cows with purified K99 antigen, K99+ anucleated live E. coli, and whole cell bacterins containing enterotoxigenic E. coli for prevention of enterotoxigenic colibacillosis of calves. 443-455. https://www.cabdirect.org/cabdirect/abstract/19800463121

Ohashi, S., Shiba, F., Haga, Y., Ajito, T., Yamada, Y., Nemoto, H., & Motoyoshi, S. (1990). Passive immunizing effect of neonatal calves by vaccinating dams with Escherichia coli bacterin containing K99 antigen against experimental colibacillosis. *Bulletin of Nippon Veterinary and Animal Science University*(No. 39), 40-49. https://www.cabdirect.org/cabdirect/abstract/19952205080

Daigneault, J., Thurmond, M., Anderson, M., Tyler, J., Picanso, J., & Cullor, J. (1991). Effect of vaccination with the R mutant Escherichia coli (J5) antigen on morbidity and mortality of dairy calves. *Am J Vet Res, 52*(9), 1492-1496.

Cornaglia, E. M., Fernandez, F. M., Gottschalk, M., Barrandeguy, M. E., Luchelli, A., Pasini, M. I., Saif, L. J., Parraud, J. R., Romat, A., & Schudel, A. A. (1992). Reduction in morbidity due to diarrhea in nursing beef calves by use of an inactivated oil-adjuvanted rotavirus-Escherichia coli vaccine in the dam. *Vet Microbiol, 30*(2-3), 191-202. <https://doi.org/10.1016/0378-1135(92)90113-8>

Avila, F. A., Paulillo, A. C., Schocken-Iturrino, R. P., Lucas, F. A., Orgaz, A., & Quintana, J. L. (1995). A comparative study of the efficiency of a pro-biotic and the anti-K99 and anti-A14 vaccines in the control of diarrhea in calves in Brazil. *Rev Elev Med Vet Pays Trop, 48*(3), 239-243.

Selim, S. A., Cullor, J. S., Smith, B. P., Blanchard, P., Farver, T. B., Hoffman, R., Dilling, G., Roden, L. D., & Wilgenburg, B. (1995). The effect of Escherichia coli J5 and modified live Salmonella dublin vaccines in artificially reared neonatal calves. *Vaccine, 13*(4), 381-390. https://doi.org/10.1016/0264-410x(95)98262-9.

Yano, T., Garcia, M., Leite, D. S., Pestana-de-Castro, A. F., & Shenk, M. A. (1995). Determination of the efficiency of K99-F41 fimbrial antigen vaccine in newborn calves. *Braz J Med Biol Res, 28*(6), 651-654.

Kohara, J., Hirai, T., Mori, K., Ishizaki, H., & Tsunemitsu, H. (1997). Enhancement of passive immunity with maternal vaccine against newborn calf diarrhea. *J Vet Med Sci, 59*(11), 1023-1025. https://doi.org/10.1292/jvms.59.1023.

Bendali, F., Sanaa, M., Bichet, H., & Schelcher, F. (1999). Risk factors associated with diarrhoea in newborn calves. *Vet Res, 30*(5), 509-522.

Perk, K., Moussa, A. A., Tromp, A. M., Reda, I. M., Refai, M., Friedman, A., Farid, A. F., Gallily, O., Salah, S. M., & Saif, L. (2000). Neonatal diarrheal disease of dairy cattle in Egypt and Israel. *Israel Journal of Veterinary Medicine, 55*(1), 13-18. https://www.cabdirect.org/cabdirect/abstract/20002216311

Le Rousic, S., Klein, N., Houghton, S., & Charleston, B. (2000). Use of colostrum from rotavirus-immunised cows as a single feed to prevent rotavirus-induced diarrhoea in calves. *Vet Rec, 147*(6), 160-161. https://doi.org/10.1136/vr.147.6.160.

Jayappa, H., Davis, R., Dierks, L., Sweeney, D., & Wasmoen, T. (2008). Demonstration of passive protection in neonatal calves against colibacillosis following immunization of pregnant heifers at 3 months of gestation. *Vet Ther, 9*(4), 283-289.

Younis, E. E., Ahmed, A. M., El-Khodery, S. A., Osman, S. A., & El-Naker, Y. F. (2009). Molecular screening and risk factors of enterotoxigenic Escherichia coli and Salmonella spp. in diarrheic neonatal calves in Egypt. *Res Vet Sci, 87*(3), 373-379. https://doi.org/10.1016/j.rvsc.2009.04.006. Epub 2009 May 5.

Meganck, V., Hoflack, G., Piepers, S., & Opsomer, G. (2015). Evaluation of a protocol to reduce the incidence of neonatal calf diarrhoea on dairy herds. *Prev Vet Med, 118*(1), 64-70. https://doi.org/10.1016/j.prevetmed.2014.11.007. Epub 2014 Nov 15.

Rocha, T. G., Silva, F. D., Gregori, F., Alfieri, A. A., Buzinaro, M. D., & Fagliari, J. J. (2017). Longitudinal study of bovine rotavirus group A in newborn calves from vaccinated and unvaccinated dairy herds. *Trop Anim Health Prod, 49*(4), 783-790. https://doi.org/10.1007/s11250-017-1263-2. Epub 2017 Mar 20.

**Salmonella vaccine studies**

Smith, H. W. (1965). The Immunization of Mice, Calves and Pigs Against Salmonella Dublin and Salmonella Cholerae-Suis Infections. *J Hyg (Lond), 63*, 117-135. https://doi.org/10.1017/s0022172400045022.

Rankin, J. D., Taylor, R. J., & Newman, G. (1967). The protection of calves against infection with Salmonella typhimurium by means of a vaccine prepared from Salmonella dublin (strain 51). *Vet Rec, 80*(25), 720-726. https://doi.org/10.1136/vr.80.25.720.

Rankin, J. D., & Taylor, R. J. (1970). An attempt passively to immunise calves against salmonella infection by vaccination of their dams. *Vet Rec, 86*(9), 254-256. https://doi.org/10.1136/vr.86.9.254.

Bairey, M. H. (1978). Immunization of calves against salmonellosis. *J Am Vet Med Assoc, 173*(5 Pt 2), 610-613.

Smith, B. P., Habasha, F. G., Reina-Guierra, M., & Hardy, A. J. (1980). Immunization of calves against salmonellosis. *Am J Vet Res, 41*(12), 1947-1951.

Chaturvedi, G. C., & Sharma, V. K. (1981). Cell-mediated immunoprotection in calves immunized with rough Salmonella dublin. *Br Vet J, 137*(4), 421-430.

Aitken, M. M., Brown, G. T., Jones, P. W., & Collins, P. (1983). Salmonella saint-paul infection in calves. *J Hyg (Lond), 91*(2), 259-265. https://doi.org/10.1017/s0022172400060265.

Robertsson, J. A., Lindberg, A. A., Hoiseth, S., & Stocker, B. A. (1983). Salmonella typhimurium infection in calves: protection and survival of virulent challenge bacteria after immunization with live or inactivated vaccines. *Infect Immun, 41*(2), 742-750.

Stocker, B. A., Hoiseth, S. K., & Smith, B. P. (1983). Aromatic-dependent "Salmonella sp." as live vaccine in mice and calves. *Dev Biol Stand, 53*, 47-54.

Smith, B. P., Reina-Guerra, M., Stocker, B. A., Hoiseth, S. K., & Johnson, E. (1984). Aromatic-dependent Salmonella dublin as a parenteral modified live vaccine for calves. *Am J Vet Res, 45*(11), 2231-2235.

Clarke, R. C., & Gyles, C. L. (1987). Vaccination of calves with a diaminopimelic acid mutant of Salmonella typhimurium. *Can J Vet Res, 51*(1), 32-38.

Peters, A. R., Wray, C., & Allsup, T. N. (1987). Serological response of calves to a dead salmonella vaccine and its relation to live weight and performance. *Vet Rec, 121*(4), 84-85. https://doi.org/10.1136/vr.121.4.84.

Wray, C., & McLaren, I. (1987). Further studies on the use of Gal E mutants of Salmonella typhimurium in calves: oral vaccination and toxicity studies. *Zentralbl Veterinarmed B, 34*(1), 22-29. https://doi.org/10.1111/j.1439-0450.1987.tb00366.x.

Jones, P. W., Collins, P., & Aitken, M. M. (1988). Passive protection of calves against experimental infection with Salmonella typhimurium. *Vet Rec, 123*(21), 536-541. https://doi.org/10.1136/vr.123.21.536.

Mikula, I., Rosocha, J., & Pilipcinec, E. (1989). Immunization of calves with live and inactivated whole-cell vaccines against Salmonella typhimurium infection. *Acta Vet Hung, 37*(3), 219-226.

Anderson, J., Smith, B. P., & Ulrich, J. T. (1991). Vaccination of calves with a modified bacterin or oil-in-water emulsion containing alkali-detoxified Salmonella typhimurium lipopolysaccharide. *Am J Vet Res, 52*(4), 596-601.

Jones, P. W., Dougan, G.krogh, Hayward, C., Mackensie, N., Collins, P., & Chatfield, S. N. (1991). Oral vaccination of calves against experimental salmonellosis using a double aro mutant of Salmonella typhimurium. *Vaccine, 9*(1), 29-34. https://doi.org/10.1016/0264-410x(91)90313-u.

Mukkur, T. K., Walker, K. H., Jones, D., Wronski, E., & Love, D. N. (1991). Immunizing efficacy of aromatic-dependent Salmonella dublin in mice and calves. *Comp Immunol Microbiol Infect Dis, 14*(3), 243-256. https://doi.org/10.1016/0147-9571(91)90005-x.

Mikula, I., Pistl, J., & Rosocha, J. (1992). Dialyzable leukocyte extract used in the prevention of Salmonella infection in calves. *Vet Immunol Immunopathol, 32*(1-2), 113-124. https://doi.org/10.1016/0165-2427(92)90073-y.

Smith, B. P., Dilling, G. W., Da Roden, L., & Stocker, B. A. (1993). Vaccination of calves with orally administered aromatic-dependent Salmonella dublin. *Am J Vet Res, 54*(8), 1249-1255.

Selim, S. A., Cullor, J. S., Smith, B. P., Blanchard, P., Farver, T. B., Hoffman, R., Dilling, G., Roden, L. D., & Wilgenburg, B. (1995). The effect of Escherichia coli J5 and modified live Salmonella dublin vaccines in artificially reared neonatal calves. *Vaccine, 13*(4), 381-390. https://doi.org/10.1016/0264-410x(95)98262-9.

House, J. K., Ontiveros, M. M., Blackmer, N. M., Dueger, E. L., Fitchhorn, J. B., McArthur, G. R., & Smith, B. P. (2001). Evaluation of an autogenous Salmonella bacterin and a modified live Salmonella serotype Choleraesuis vaccine on a commercial dairy farm. *Am J Vet Res, 62*(12), 1897-1902. https://doi.org/10.2460/ajvr.2001.62.1897.

Van der Walt, M. L., Vorster, J. H., Steyn, H. C., & Greeff, A. S. (2001). Auxotrophic, plasmid-cured Salmonella enterica serovar typhimurium for use as a live vaccine in calves. *Vet Microbiol, 80*(4), 373-381. https://doi.org/10.1016/s0378-1135(01)00325-x.

Dueger, E. L., House, J. K., Heithoff, D. M., & Mahan, M. J. (2003). Salmonella DNA adenine methylase mutants elicit early and late onset protective immune responses in calves. *Vaccine, 21*(23), 3249-3258. https://doi.org/10.1016/s0264-410x(03)00252-4.

Mohler, V. L., Heithoff, D. M., Mahan, M. J., Walker, K. H., Hornitzky, M. A., McConnell, C. S., Shum, L. W., & House, J. K. (2006). Cross-protective immunity in calves conferred by a DNA adenine methylase deficient Salmonellaenterica serovar Typhimurium vaccine. *Vaccine, 24*(9), 1339-1345. https://doi.org/10.1016/j.vaccine.2005.09.022. Epub 2005 Oct 5.

Mizuno, T., McLennan, M., & Trott, D. (2008). Intramuscular vaccination of young calves with a Salmonella Dublin metabolic-drift mutant provides superior protection to oral delivery. *Vet Res, 39*(3), 26. https://doi.org/10.1051/vetres:2008001. Epub 2008 Feb 15.

Mohler, V. L., Heithoff, D. M., Mahan, M. J., Walker, K. H., Hornitzky, M. A., Shum, L. W., Makin, K. J., & House, J. K. (2008). Cross-protective immunity conferred by a DNA adenine methylase deficient Salmonella enterica serovar Typhimurium vaccine in calves challenged with Salmonella serovar Newport. *Vaccine, 26*(14), 1751-1758. https://doi.org/10.1016/j.vaccine.2008.01.018. Epub 2008 Feb 4.

Habing, G. G., Neuder, L. M., Raphael, W., Piper-Youngs, H., & Kaneene, J. B. (2011). Efficacy of oral administration of a modified-live Salmonella Dublin vaccine in calves. *J Am Vet Med Assoc, 238*(9), 1184-1190. https://doi.org/10.2460/javma.238.9.1184.

Foster, D., Jacob, M., Stowe, D., & Smith, G. (2019). Exploratory cohort study to determine if dry cow vaccination with a Salmonella Newport bacterin can protect dairy calves against oral Salmonella challenge. *J Vet Intern Med, 33*(4), 1796-1806. https://doi.org/10.1111/jvim.15529. Epub 2019 May 27.

**Vaccine studies for other pathogens**

**Bovine Viral Diarrhea virus**

Bolin, S. R., & Ridpath, J. F. (1996). Glycoprotein E2 of bovine viral diarrhea virus expressed in insect cells provides calves limited protection from systemic infection and disease. *Arch Virol, 141*(8), 1463-1477. https://doi.org/10.1007/bf01718248.

Makoschey, B., Janssen, M. G., Vrijenhoek, M. P., Korsten, J. H., & Marel, P. (2001). An inactivated bovine virus diarrhoea virus (BVDV) type 1 vaccine affords clinical protection against BVDV type 2. *Vaccine, 19*(23-24), 3261-3268. https://doi.org/10.1016/s0264-410x(01)00003-2.

Hamers, C., Couvreur, B., Dehan, P., Letellier, C., Fischer, L., Brun, A. J., Lewalle, P., Michaux, C., Pastoret, P. P., & Kerkhofs, P. (2003). Assessment of the clinical and virological protection provided by a commercial inactivated bovine viral diarrhoea virus genotype 1 vaccine against a BVDV genotype 2 challenge. *Vet Rec, 153*(8), 236-240. https://doi.org/10.1136/vr.153.8.236.

Chamorro, M. F., Walz, P. H., Passler, T., van Santen, E., Gard, J., Rodning, S. P., Riddell, K. P., Galik, P. K., & Zhang, Y. (2015). Efficacy of multivalent, modified- live virus (MLV) vaccines administered to early weaned beef calves subsequently challenged with virulent Bovine viral diarrhea virus type 2. *BMC Vet Res, 11*, 29. https://doi.org/10.1186/s12917-015-0342-8.

Bittar, J. H. J., Hoyos-Jaramillo, A., Hurley, D. J., Woolums, A. R., Havenga, L. J., Lourenco, J. M., Barnett, G., Gomes, V., Saliki, J. T., Harmon, D. D., & Palomares, R. A. (2018). Effects of injectable trace minerals administered concurrently with a modified live virus vaccine on long-term protection against bovine viral diarrhea virus acute infection in dairy calves. *Res Vet Sci, 119*, 250-258. https://doi.org/10.1016/j.rvsc.2018.07.003. Epub 2018 Jul 21.

Chen, C., Liu, H., Meng, Q., Xia, M., & Wu, H. (2020). Evaluation of protection against bovine viral diarrhea virus type 2 after vaccination of the calves with bovine viral diarrhea virus type 1 combo inactivate vaccine. *Arq. Bras. Med. Vet. Zoot., 72*(3), 655-663.

**Cryptrosporidium**

Fayer, R., Andrews, C., Ungar, B. L. P., & Blagburn, B. (1989). Efficacy of hyperimmune bovine colostrum for prophylaxis of cryptosporidiosis in neonatal calves. *Journal of Parasitology, 75*(3), 393-397. https://www.cabdirect.org/cabdirect/abstract/19890859022

Harp, J. A., & Goff, J. P. (1995). Protection of calves with a vaccine against Cryptosporidium parvum. *J Parasitol, 81*(1), 54-57.

Harp, J. A., Jardon, P., Atwill, E. R., Zylstra, M., Checel, S., Goff, J. P., & De Simone, C. (1996). Field testing of prophylactic measures against Cryptosporidium parvum infection in calves in a California dairy herd. *Am J Vet Res, 57*(11), 1586-1588.

Perryman, L. E., Kapil, S. J., Jones, M. L., & Hunt, E. L. (1999). Protection of calves against cryptosporidiosis with immune bovine colostrum induced by a Cryptosporidium parvum recombinant protein. *Vaccine, 17*(17), 2142-2149. https://doi.org/10.1016/s0264-410x(98)00477-0.

Askari, N., Shayan, P., Mokhber-Dezfouli, M. R., Ebrahimzadeh, E., Lotfollahzadeh, S., Rostami, A., Amininia, N., & Ragh, M. J. (2016). Evaluation of recombinant P23 protein as a vaccine for passive immunization of newborn calves against Cryptosporidium parvum. *Parasite Immunol, 38*(5), 282-289. https://doi.org/10.1111/pim.12317.

**Giardia**

Uehlinger, F. D., O'Handley, R. M., Greenwood, S. J., Guselle, N. J., Gabor, L. J., Van Velsen, C. M., Steuart, R. F., & Barkema, H. W. (2007). Efficacy of vaccination in preventing giardiasis in calves. *Vet Parasitol, 146*(1-2), 182-188. https://doi.org/10.1016/j.vetpar.2007.01.013. Epub 2007 Mar 12.

**Norovirus**

Han, M. G., Cheetham, S., Azevedo, M., Thomas, C., & Saif, L. J. (2006). Immune responses to bovine norovirus-like particles with various adjuvants and analysis of protection in gnotobiotic calves. *Vaccine, 24*(3), 317-326. https://doi.org/10.1016/j.vaccine.2005.07.071. Epub 2005 Aug 10.

**Parvovirus**

Kohara, J., Hirai, T., Mori, K., Ishizaki, H., & Tsunemitsu, H. (1997). Enhancement of passive immunity with maternal vaccine against newborn calf diarrhea. *J Vet Med Sci, 59*(11), 1023-1025. https://doi.org/10.1292/jvms.59.1023.

**Studies evaluating no specific pathogen**

Frank, N. A., & Kaneene, J. B. (1993). Management risk factors associated with calf diarrhea in Michigan dairy herds. *J Dairy Sci, 76*(5), 1313-1323. https://doi.org/10.3168/jds.S0022-0302(93)77462-7.
